# Motor imagery-based brain-computer interfaces: an exploration of multiclass motor imagery-based control for Emotiv EPOC X

**DOI:** 10.3389/fninf.2025.1625279

**Published:** 2025-08-12

**Authors:** Paulina Tarara, Iwona Przybył, Julius Schöning, Artur Gunia

**Affiliations:** ^1^Multigraphical Creation Studio, Academy of Fine Arts and Design in Katowice, Katowice, Poland; ^2^Business Service Galop, Katowice, Poland; ^3^Faculty of Engineering and Computer Science, Osnabrück University of Applied Sciences, Osnabrück, Germany; ^4^Centre for Cognitive Science, Jagiellonian University, Kraków, Poland

**Keywords:** brain-computer interface (BCI), Emotiv EPOC X, motor imagery, body awareness training, lateral bending

## Abstract

**Introduction:**

Enhancing the command capacity of motor imagery (MI)-based brain-computer interfaces (BCIs) remains a significant challenge in neuroinformatics, especially for real-world assistive applications. This study explores a multiclass BCI system designed to classify multiple MI tasks using a low-cost EEG device.

**Methods:**

A BCI system was developed to classify six mental states: resting state, left and right hand movement imagery, tongue movement, and left and right lateral bending, using EEG data collected with the Emotiv EPOC X headset. Seven participants underwent a body awareness training protocol integrating mindfulness and physical exercises to improve MI performance. Machine learning techniques were applied to extract discriminative features from the EEG signals.

**Results:**

Post-training assessments indicated modest improvements in participants' MI proficiency. However, classification performance was limited due to inter- and intra-subject signal variability and the technical constraints of the consumer-grade EEG hardware.

**Discussion:**

These findings highlight the value of combining user training with MI-based BCIs and the need to optimize signal quality for reliable performance. The results support the feasibility of scalable, multiclass MI paradigms in low-cost, user-centered neurotechnology applications, while pointing to critical areas for future system enhancement.

## 1 Introduction

Brain-computer interface (BCI) technology enables users to interact with and control their environment using neural activity alone. When fully developed, BCI systems have potential applications in a wide range of domains, including ergonomics at work (Ekandem et al., 2012; Venthur et al., 2010), the arts (Wadeson et al., 2015; Gürkök and Nijholt, 2013), smart environments (Tabbal et al., 2018), neurotherapy (Tabernig et al., 2018; Zhuang et al., 2020), and neuroprosthetics (Cajigas et al., 2021; Hayashibe et al., 2015). Specifically, BCI could be used to monitor and regulate cognitive states in employees or students, helping determine whether an individual should engage in a cognitively demanding task or take a break (Gerjets et al., 2014). In addition, it offers communication solutions for people with paralysis and can facilitate the control of smart home systems (Birbaumer and Cohen, 2007). In entertainment and virtual environments, BCIs could enable users to control characters in video games or avatars in virtual reality (Wen et al., 2021). Despite its remarkable potential and significant research over the last 30 years, the widespread adoption and implementation of BCI technology remains limited.

The limited adoption of BCI in daily life is due to three key challenges. First, active BCIs, which rely on voluntary modulation of brain activity, generally provide only two to three commands, much fewer than traditional input devices, such as keyboards or mice. Second, controlling an active BCI is challenging, with up to one-third of users unable to generate the necessary brain signals, requiring extensive training. Third, brain activity detection requires specialized hardware, and although commercial options are emerging, they still require further validation.

The study explored a new possibility of expanding the number of classes in mental task-based BCI by combining the resting state with motor imagery (MI) of five movements: left hand movement, right hand movement, tongue movement, left and right lateral bending. Although previously studied separately, such a combination of imagined movements and resting state was never implemented in a MI-based BCI. The present study utilized the mobile electroencephalography (EEG) device *Emotiv EPOC X*, classification-based machine learning techniques, and a novel procedure designed to train MI.

## 2 Motor imagery-based brain-computer interface

MI-based BCIs enable users to control systems through imagined movements, offering a non-invasive, stimulus-independent communication method. By detecting changes in sensorimotor rhythms (SMRs) via EEG, these systems translate mental tasks into commands, making them valuable for assistive and neuroadaptive technologies.

### 2.1 State of the art on motor imagery-based brain-computer interface

The term “Brain-Computer Interface” (BCI) was coined by (Vidal 1977) in the 1970s, demonstrating real-time processing of EEG signals and control of a cursor using visual-evoked potentials. The first attempt to read one's cognitive state began with Albertino Mosso's studies on mental activity and blood circulation in the late 19th century (Poldrack, 2018), and Hans Berger's development of electroencephalography in the 1920s. The most accepted definition of BCI combines the neuroscientific perspective of (Wolpaw and Wolpaw 2012) with the cognitive perspective of (Zander and Kothe 2011), describing BCI as a system that measures the activity of the central nervous system (CNS) to predict the cognitive state of the user and produce an artificial output that influences the interaction between the CNS and the environment and provides feedback to the user.

There are several paradigms for acquiring BCI input, the most widely used of which are based on physiological brain activity. These include steady-state visually evoked potentials (SSVEPs), event-related potential (ERP) on P3b, sensorimotor rhythms (SMRs), and slow cortical potentials (SCPs). SSVEPs are evoked by fast and repetitive stimuli, such as flickering lights, that modulate the frequency and voltage of the ongoing oscillations in the corresponding areas of the occipital cortex in such a way that the frequency of these oscillations matches the frequency of presentation of the given stimulus (Allison et al., 2012). In P3-based BCI, the component P3b, which is a positive change in potential appearing around 300 ms after stimulus presentation over central parietal sites on the scalp, is evoked in an odd-ball paradigm by a task-related infrequent stimulus (Sellers et al., 2012). SCPs (Allison et al., 2012) and SMRs (Pfurtscheller and McFarland, 2012), resulting from oscillatory changes in the sensorimotor cortex during motor preparation or imagery, are analyzed in the time and frequency domains, respectively. Both the P3 and SSVEP paradigms are reactive and require minimal user training, but they are based on external stimuli. In contrast, the SCP and SMR paradigms are independent of external stimuli but often require extensive and fatiguing user training.

Mental imagery-based BCIs rely on users performing cognitive tasks to generate control signals. Such tasks include mental rotation, counting, face or speech imagery, auditory and olfactory imagery, spatial navigation, self-induced emotions, and MI (Roc et al., 2021), the most commonly studied. MI encompasses various imagined actions, typically involving simple body movements such as movements of the right or left hand (Arias-Mora et al., 2015), feet (Bousseta et al., 2018), or tongue (Lin and Lo, 2016). Less frequently studied movements include swallowing (Yang et al., 2014), lateral bending (Li et al., 2019), and sign language (AlQattan and Sepulveda, 2017). Many studies do not specify the exact nature of the imagined movements.

Neuronal populations in the cortex that correlate with MI express oscillatory activity called Sensorimotor Rhythms (SMR) (Pfurtscheller and Lopes da Silva, 1999). Depending on the frequency band, three types of SMR were identified: the mu rhythm with lower mu 7–10 Hz and higher mu 10–12 Hz, the beta rhythm with lower beta 12–20 Hz and higher beta 20–30 Hz and the gamma rhythm with 30–200 Hz (Pfurtscheller and McFarland, 2012). In the field of BCI studies, the specific boundaries of these bands differ between studies (Jeunet et al., 2019). Moreover, since most BCIs employ EEG, which does not precisely measure gamma, the focus is placed mainly on mu and beta rhythms (Jeunet et al., 2019).

### 2.2 Machine learning for motor imagery-based brain-computer interface

Machine learning is crucial for BCI systems because it enables the accurate interpretation of complex brain signals, converting them into actionable commands. It helps distinguish subtle patterns in neural data, allowing BCIs to adapt to individual users' brain activity. Machine learning in BCI consists of the following stages: signal acquisition, signal processing, classification, and performance evaluation.

#### 2.2.1 Signal acquisition

Signal acquisition for BCI involves collecting a biosignal from the cerebral cortex using electrodes placed on the scalp, typically following the 10–20 system for optimal electrode placement. Choosing the right electrodes, particularly those over the primary motor cortex and primary somatosensory cortex (Schnitzler et al., 1997) and the supplementary motor area and premotor areas (Dechent et al., 2004), improves classification accuracy and machine learning speed, with research indicating that the use of too many or too few electrodes results in poor performance and the use of 8 to 36 electrodes (Tam et al., 2011) yields the best results for real-time applications such as operating a wheelchair or facilitating communication.

#### 2.2.2 Signal processing

In BCI devices, signal processing is divided into the following parts: preprocessing, feature selection, and/or extraction and classification.

**Preprocessing** such as temporal filtering, removes these artifacts to enhance the signal-to-noise ratio, ensuring that relevant data are processed for better classification accuracy (Aggarwal and Chugh, 2019). Preprocessing is important since EEG signals include activity in the cerebral cortex and artifacts of eye movements, blinking, head movements, jaw clenching, and external stimuli. In MI-based BCI devices, particular attention is given to SMR waves, specifically mu waves in the 7–13 Hz frequency range (Pfurtscheller and Neuper, 2001), using filters like Butterworth and Chebyshev to refine the signal (Singh et al., 2021).

**Feature selection** is the method that removes redundant information to reduce the dimensionality of vast amount of EEG data and thus improve its accuracy (Singh et al., 2021). Feature selection methods, which will enhance classifier performance and speed, can be categorized into filter methods—independent of the classifier with low computational cost, wrapper methods—evaluating data subsets for specific classifiers, and embedded methods—integrating feature selection into classifier training (Guyon and Elisseeff, 2003).

**Feature extraction** transforms preprocessed EEG data into a new feature space to reduce data volume for better classifier performance and faster computation. Six categories of feature extraction methods include: time domain, spectral domain, time-frequency domain, spatial domain, spatio-temporal domain, and the Riemannian Manifold method, each focusing on different aspects of the EEG signal to obtain relevant features for analysis (Singh et al., 2021).

**Classification** involves interpreting brain activity and converting it into actionable commands using algorithms trained on labeled data to predict target variables based on extracted features. (Lotte et al. 2007) categorizes classifiers into generative-discriminative, static-dynamic, stable-unstable, and regularized types, with popular algorithms including linear classifiers such as linear discriminant analysis (LDA) and support vector machine (SVM), artificial neural networks (ANN), non-linear Bayesian classifiers, and k-nearest neighbor (KNN) classifiers.

Improving classifier performance and accuracy can involve combining different classifiers, reducing data dimensions through feature selection and extraction, and optimizing algorithm hyperparameters. Tools such as GridSearch can help identify the optimal set of hyperparameters by exhaustively searching and evaluating different combinations of parameters to maximize the precision of a specific data set (Syarif et al., 2016; Feurer and Hutter, 2019).

#### 2.2.3 Performance evaluation

Performance evaluation is crucial to assess the BCI operation, ensure the classification algorithm's effectiveness, and verify that the results are not due to chance. Evaluation involves considering the number of trials and the likelihood of class occurrence, with the actual chance level varying based on the number of classes, e.g., a two-class problem has a chance level 70% instead of 50% (Müller-Putz et al., 2008). Standard evaluation metrics for BCI classification include accuracy, Cohen's Kappa coefficient, Confusion Matrix, specificity, and sensitivity. These metrics help determine how well the BCI performs and if the results are statistically significant.

### 2.3 Brain-computer interface applications

BCI applications translate brainwave modulation into actions, bypassing neuromuscular output to enable control of devices such as wheelchairs and communication tools for individuals with motor impairments. BCIs serve medical and nonmedical purposes, helping people with disabilities by replacing, restoring, or enhancing neuromuscular functions (Wolpaw and Wolpaw, 2012; Bamdad et al., 2015; Värbu et al., 2022), while also offering applications in monitoring, device control, and entertainment (van Erp et al., 2012; Kutt et al., 2015; Zioga et al., 2018; Douibi et al., 2021; Värbu et al., 2022). Despite their potential, achieving consistent accuracy and user experience is challenging outside controlled laboratory settings (Hammer et al., 2012; Wolpaw and Wolpaw, 2012; Mridha et al., 2021).

### 2.4 Brain-computer interface study objectives

This study aimed to develop an MI-based BCI system to improve communication for people who have lost neuromuscular function due to injury or disease. To make BCI a viable daily communication tool for individuals with disabilities, it must be user-friendly, independent, and multiclass. Commercial EEG headsets, such as the *Emotiv EPOC X*, offer a portable and cost-effective alternative (Mwata-Velu et al., 2021) to medical-grade, while providing high-quality signals.

This study explored whether an MI-based BCI using the *Emotiv EPOC X* could accurately distinguish between six classes: resting state and five imagined movements: left hand, right hand, tongue movement, left and right lateral bending. Participants were familiarized with the BCI, underwent data acquisition and training, and tested their ability to use the BCI by playing the song “Soft Kitty”^1^ with success determined by classification results above the chance level. The selected movements are novel, with previous research indicating that left- and right-lateral bending can be distinguished using the *Emotiv EPOC X* headset (Li et al., 2019).

## 3 Materials and methods

This section outlines the experimental design, tools, and procedures for developing and evaluating the BCI system. It describes the architecture of the BCI framework, the hardware and software employed, participant details, training protocols, data acquisition process, and the machine learning pipeline used for signal processing and classification. Each component was carefully selected and integrated to ensure reliable performance and user engagement throughout the study.

### 3.1 Architecture

**Figure 1:**
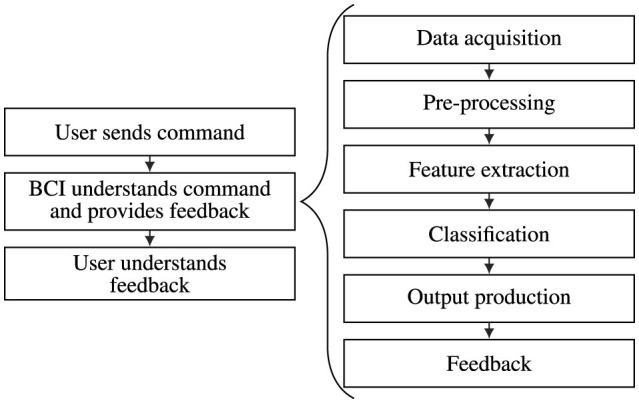
Combination of the two main architectures of a BCI system. The three stages of the architecture, which focus is put on the interaction between two entities of the BCIs—the user and the computer—are presented on the left. The six architecture steps centered on the BCI work cycle, which constitute the second stage of the more general architecture, are presented on the right.

The typical BCI architecture consists of six key stages: CNS activity collection, offline preprocessing, feature extraction, classification, real-time data analysis, and feedback delivery based on predicted mental states. These stages form the core of a broader BCI framework, including user training to modulate CNS activity for computer recognition, enabling feedback that enhances control of brain activity. Figure 1 shows the architecture used in this study.

### 3.2 Emotiv Epoc X

**Figure 2:**
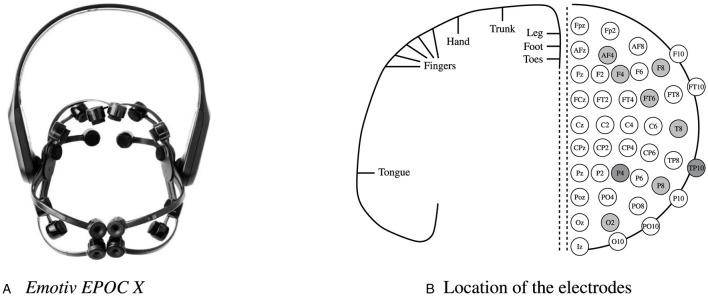
The commercial EEG headset Emotiv EPOC X **(A)** and its 14 channels: AF3, F7, F3, FC5, T7, P7, O1, O2, P8, T8, FC6, F4, F8, AF4. The motor homunculus with electrode locations in the *Emotiv EPOC X* headset. The location of the electrodes **(B)** according to the international 10–20 system. Light gray: sensors. Dark gray: references.

In nonclinical contexts, commercial EEG kits^2^ are gaining popularity for use in laboratory research, ecological studies, and the gaming industry. The *Emotiv EPOC X* was deliberately selected for this study due to its ease of use, rapid self-mounting capability, user comfort, and—critically—its demonstrated signal quality comparable to medical-grade EEG systems (Duvinage et al., 2012), cf. Figure 2A. It features 14 electrodes, 4 reference channels, and a sampling rate of 128 Hz, with wet electrodes soaked in a solution of water and sodium chloride (NaCl). The electrodes are placed according to the 10–20 system. As illustrated in Figure 2B electrodes locations are as follows: AF3, F7, F3, FC5, T7, P7, O1, O2, P8, T8, FC6, F4, F8, AF4.

### 3.3 Study participants

The study involved 7 participants (3 women and 4 men) with an average age of 24 years, ranging from 21 to 26 years. The number of participants was chosen based on previous studies of MI-based BCI built with *Emotiv EPOC X*, in which sample sizes typically ranged from 4 to 7 (Arias-Mora et al., 2015; Bousseta et al., 2018; Lin and Lo, 2016; Yue et al., 2021). Detailed participant information is presented in Table 1. Written informed consent was obtained from all participants before the study. As a token of appreciation, participants were offered a sweet snack, consisting of fruit and chocolate, after the study.

**Table 1:** Participants' data.

**ID**	**Age**	**Sex**	**Handedness**	**BCI experience**	**Playingsports**	**Others**
S01	26	Male	Right	No	Yes	Fine arts, meditation (irregularly)
S02	26	Male	Right	Yes	Yes	Fine arts, dance
S03	22	Female	Left	No	Yes	—
S04	26	Male	Right	No	Yes	—
S05	24	Female	Right	Yes	No	Dance
S06	21	Female	Right	No	Yes	Meditation
S07	21	Male	Right	No	Yes	—

Survey results presenting participants' data: age, gender, handedness, previous experience with brain-computer interfaces, sports, dance, fine arts, and meditation. (—) not provided.

### 3.4 Body awareness training

A body awareness training protocol was developed and implemented to improve the participants' ability to perform kinesthetic MI. This training combined mindfulness meditation (Eskandari and Erfanian, 2008; Jiang et al., 2021; Lo et al., 2004; Stieger et al., 2020; Tan et al., 2014, 2015) with body awareness exercises (Cassady et al., 2014)—the detailed transcript of the training can be found in the Supplementary material. Participants were guided through a 35 min video, divided into two sections, to facilitate training.

The first part was audio-based. The participants sat with their eyes closed and followed the guided instructions. The session began with an overview of the training goals, followed by a focus on breathing, a guided body scan, and a minute to do the body scan on their own.

**Figure 3:**
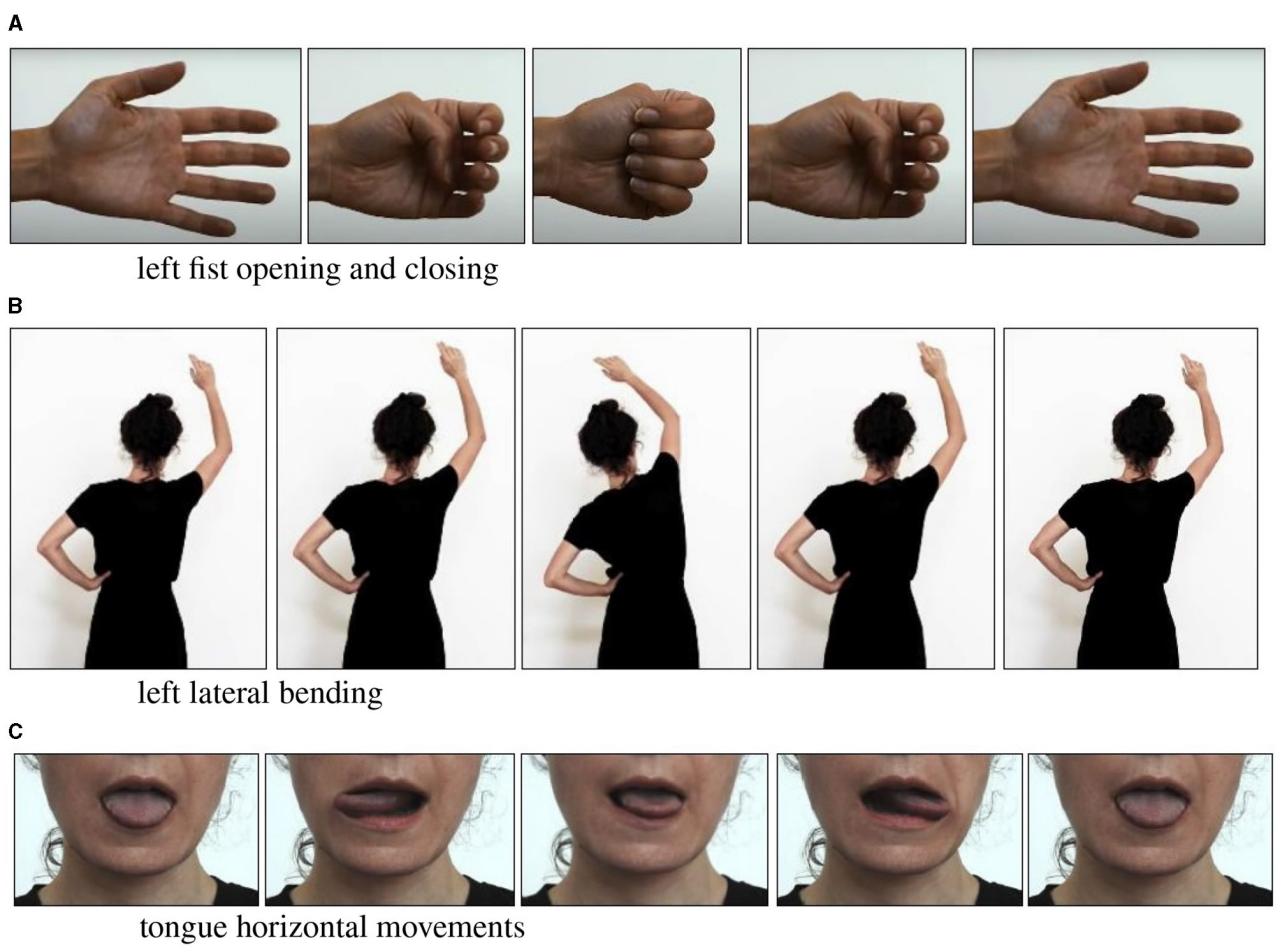
Movements presented during the body awareness training illustrate three of the five movements. Participants were instructed first to perform the movement physically **(A–C)** and then to imagine it without any physical motion. Motor imagery of the movements was used to control the built BCI.

The second part involved five audio-visual blocks, where participants stood with open eyes. Each block trained MI for one of the five movements to be used for BCI control: opening and closing the right or left fist, cf. Figure 3A, lateral bending to the right or left cf. Figure 3B, and horizontal tongue movement cf. Figure 3C. The participants were instructed on performing each movement, practiced the movement in a loop to ensure accuracy, and then focused on the movement with their eyes open. Then, they were asked to close their eyes and concentrate on the sensations associated with movement. Afterward, they stopped the physical movement and engaged in MI of the action. During MI, participants answered questions to assess the accuracy of their mental rehearsal. The video was segmented into parts, allowing participants to repeat MI as needed. Once satisfied, they proceeded to the next movement.

### 3.5 Performance measures—Movement imagery questionnaire

MI ability varies between individuals and was assessed in this study using the Movement Imagery Questionnaire (MIQ) (Hall et al., 1985; Hall and Martin, 1997; Williams et al., 2012) and the Vividness of MI Questionnaire (VMIQ) (Isaac et al., 1986; Roberts et al., 2008). The MIQ evaluates how easily participants imagine movements, using a 7-point Likert scale, while the VMIQ measures the vividness of MI, on a 5-point Likert scale.

To assess whether training improved MI skills, participants completed the Polish version of MIQ-3 (Budnik-Przybylska et al., 2016), the detailed questionnaire can be found in the Supplementary material. Since the training focused on kinesthetic MI, only two subscales were used: visual internal imagery and kinesthetic imagery, with eight items total. Participants performed a movement, then imagined it in either the kinesthetic or visual mode, rating the ease of imagery on a 7-point scale. Each participant completed the questionnaire before and after the training, with a research assistant reading the questions and recording responses.

### 3.6 Integrated brain-computer interface software

In this study, IDE Processing^3^ and OpenViBE^4^ were used. IDE Processing, a free and open-source software developed by the Processing Foundation, is designed for visual programming and the creation of digital visual art. OpenViBE is a free open-source platform that enables real-time BCI design and implementation through real-time processing of EEG signals. It supports online preprocessing, feature extraction, classification, offline analysis, and visualization of large EEG datasets. OpenViBE also provides real-time feedback and can be used to develop applications in fields such as medical applications, gaming, virtual reality, and BCI-robotics integration. It is compatible with commercial EEG devices like *Emotiv EPOC X* and OpenBCI.

**Figure 4:**
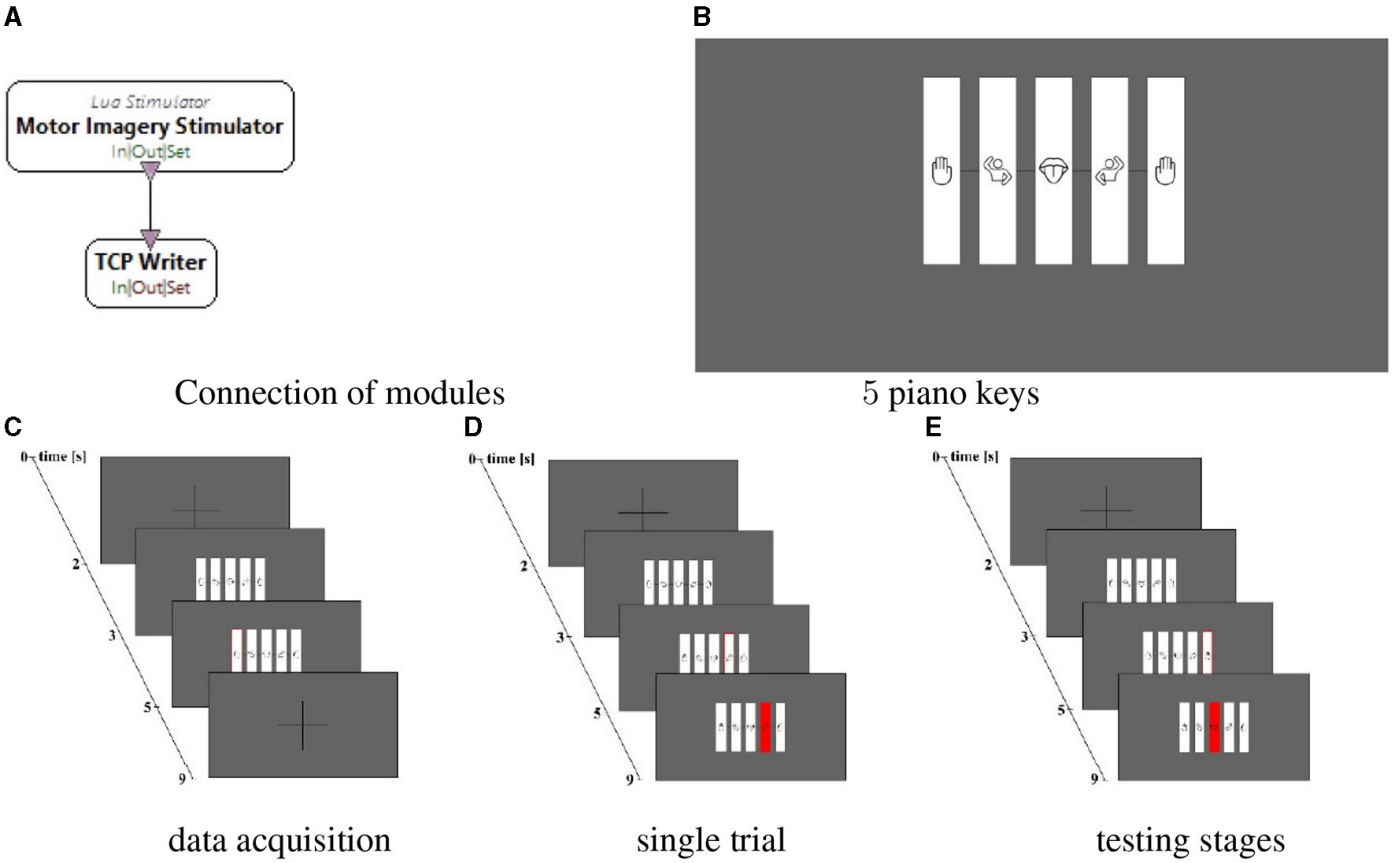
Screenshots of the application controlled by the built brain-computer interfaces (BCI). **(A)** connection of the OpenViBE Lua Stimulator box (generator of stimulations) with the OpenViBE TCP Writer box (TCP server). **(B)** 5 piano keys with the corresponding images of the body movements. **(C)** course of a single trial in the data acquisition stage. **(D)** course of a single trial in the user training and testing stages with congruent feedback. **(E)** course of a single trial in the user training and testing stages with incongruent feedback.

Virtual piano applications were designed using the above tools, shown in Figure 4B. As the maximum number of symbols to visualize with the OpenViBE Multimodal Graz Visualization box is four, the IDE Processing was used as the OpenViBE Graz Visualization box. The Lua Script provided in OpenViBE was modified to initiate the generation of the stimulations used in the study. The Lua Stimulator box read an adjusted Lua Script and sent stimulations to the transmission control protocol (TCP) Writer box, which works as a TCP server as illustrated in Figure 4A. The OpenViBE TCP Writer box sent the stimulations to the TCP socket localized in the IDE Processing script. The script reads stimuli and displays the corresponding visualization.

Participants' interaction with the BCI system consisted of three stages: data acquisition, user training, and user testing. Separate Lua and IDE Processing scripts were created for each stage.

During data acquisition, participants completed 60 trials, with 12 trials per imagined movement presented in random order. Each trial, cf. Figure 4C, began with a 2 s fixation cross, followed by the display of five keys representing movements for 1 s. A cue was presented by backlighting a single key and simultaneously playing a corresponding sound for 2*s*, marking the stimulus onset. Participants were asked to perform MI of the cued movement while the fixation cross reappeared for 4 s.

The user training stage was identical to the data acquisition (60 trials, with 12 trials per imagined movement presented in random order), except feedback which was displayed instead of the fixation cross for 4 s after the disappearance of the cue in the form of a red highlight over the predicted key and the corresponding sound. The feedback was actualized every 0.5 s and was presented as the best prediction. Examples of congruent and incongruent feedback are shown in Figures 4D, E, respectively.

The user testing stage followed the same procedure as user training, but without sound cues, and consisted of 36 trials presented in the order of the notes from the song “Soft Kitty.” The trials in all stages were separated by intervals of 1.5–3.5 s.

### 3.7 Machine learning pipeline

The theoretical analysis conducted before designing the BCI application in this study informed the selection of the following machine learning pipeline:

- Feature extraction method common spatial pattern (CSP),
- Classification method: support vector classification (SVC), i.e., SVM with a radial basis function (RBF) and a nonlinear kernel.

This study aimed to determine the optimal parameters for the SVC classifier and to identify the most effective set of electrodes to maximize classification accuracy. For this purpose, 4 sets of electrodes were included, i.e., set no. 1—(C3, C4), set no. 2—(FC5, FC6), set no. 3—(T7, FC5, F3, F4, FC6, T8), set no. 4—(T7, P3, FC5, F3, AF3, AF4, F4, FC6, P4, T8) and grid search were used to find the most optimal parameters for each of the distinguished sets of electrodes. The dataset used in this project is the MI dataset from the study by (Cho et al. 2017)^5^. A grid search, as the results for the 4 sets of electrodes can be found in Table 2, was conducted to evaluate various combinations of the following parameters:

- kernel: linear, poly, rbf,
- C: 0.01, 0.1, 0.5, 1, 3, 10, 100,
- gamma: scale, auto.

**Table 2:** Results of SVC using grid search for 4 sets of electrodes.

**Set of electrodes**	**Training set**	**Test set**	**Parameters**	**Medium accuracy**
set no. 1	0.59	0.57	C: 10, gamma: scale, kernel: rbf	0.58
set no. 2	0.59	0.57	C: 100, gamma: scale, kernel: rbf	0.57
set no. 3	0.60	0.59	C: 0.5, gamma: auto, kernel: rbf	0.59
set no. 4	0.68	0.65	C: 10, gamma: scale, kernel: rbf	0.67

5 divisions of the training set were used (k-fold = 5). The Training set column contains the accuracy results for the training set using the most optimal parameters. The Test set column contains accuracy results for the test set using the most optimal parameters selected based on the training set. The parameters column contains the most optimal parameters selected by GridSearch for each set of electrodes.

Previous analyses indicated that narrowing the bandpass frequency to 8–13 Hz and integrating the ICA, CSP, and SVC algorithms with GridSearch gives the highest accuracy for MI data. Initially, the project intended to incorporate GridSearch without relying on findings from previous studies. However, offline tests conducted before participant experiments on publicly available datasets and randomly generated oscillatory data encountered technical difficulties due to excessive computational requirements. Consequently, it was decided to omit GridSearch from the current project and instead adjust the SVC parameters for set no. 4, which comprised 10 electrodes (T7, P3, FC5, F3, AF3, AF4, F4, FC6, P4, T8). The adapted parameters are as follows: *C* = 10, gamma = scale, and kernel = RBF. The final configuration of the pipeline is as follows:

- bandpass filter 8–13 Hz,
- feature extraction method: CSP,
- classification method: SVC.

**Figure 5:**
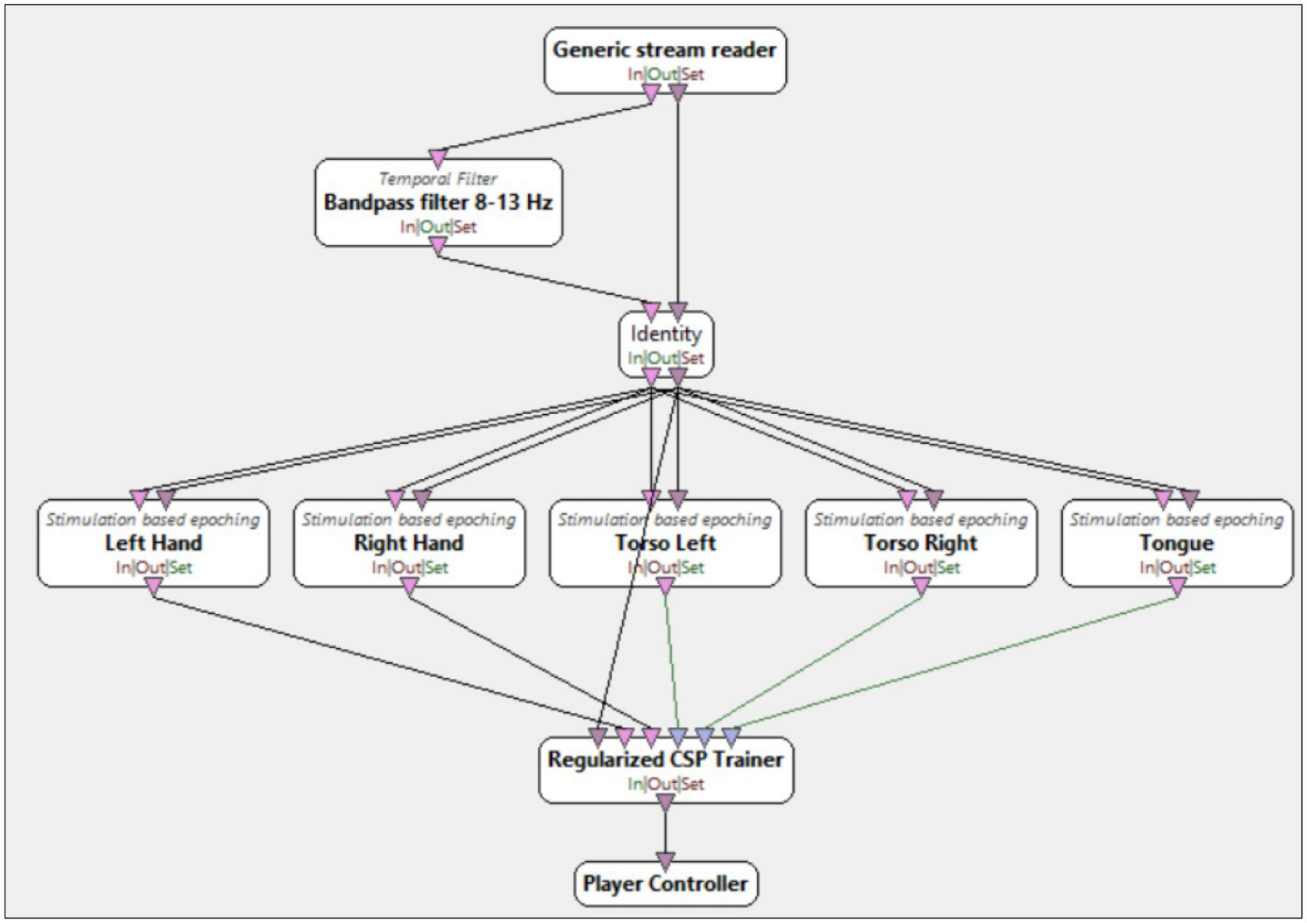
Screenshots of OpenViBE common spatial pattern (CSP) trainer algorithm. Combination of pipeline steps for training the CSP algorithm consisting of following steps: *Bandpass filter 8–13 Hz* box, *Stimulation based epoching* boxes (*Left Hand, Right Hand, Torso Left, Torso Right, Tongue*) and *Regularized CSP Trainer* box.

The classifier training process has been divided into OpenViBE CSP Trainer and OpenViBE SVM Trainer. The Pipeline OpenViBE CSP Trainer consisted of the following components, as shown in Figure 5:

- bandpass filter 8–13 Hz cf. *Temporal filter* box named *Bandpass filter 8–13 Hz*,
- division of data into 5 classes corresponding to the imagery movements cf. *Stimulation based epoching* boxes named accordingly to the imagery movement task performed by participants of this research: *Left Hand, Right Hand, Torso Left, Torso Right, Tongue*, and
- training of the CSP algorithm cf. *Regularized CSP Trainer* box.

This pipeline was designed to train the CSP algorithm to increase the variance of each of the five classes by dividing the training dataset into five subsets corresponding to each class. The dataset used for classification was divided into a training set and a test set. In this project, the training dataset was derived from the signal acquisition stage, and both CSP and SVC were trained on this data. The test set comprised data from both user training and user testing. A bandpass filter of 8–13 Hz, corresponding to the mu frequency, was applied to reduce redundant data. Epochs were defined as the 0–500 ms window following stimulus onset, which was marked by a 2-s cue involving the backlighting of a single key and the simultaneous playback of a corresponding sound. The study defined five classes corresponding to the following body movements: right hand, left hand, right torso, left torso, and tongue.

**Figure 6:**
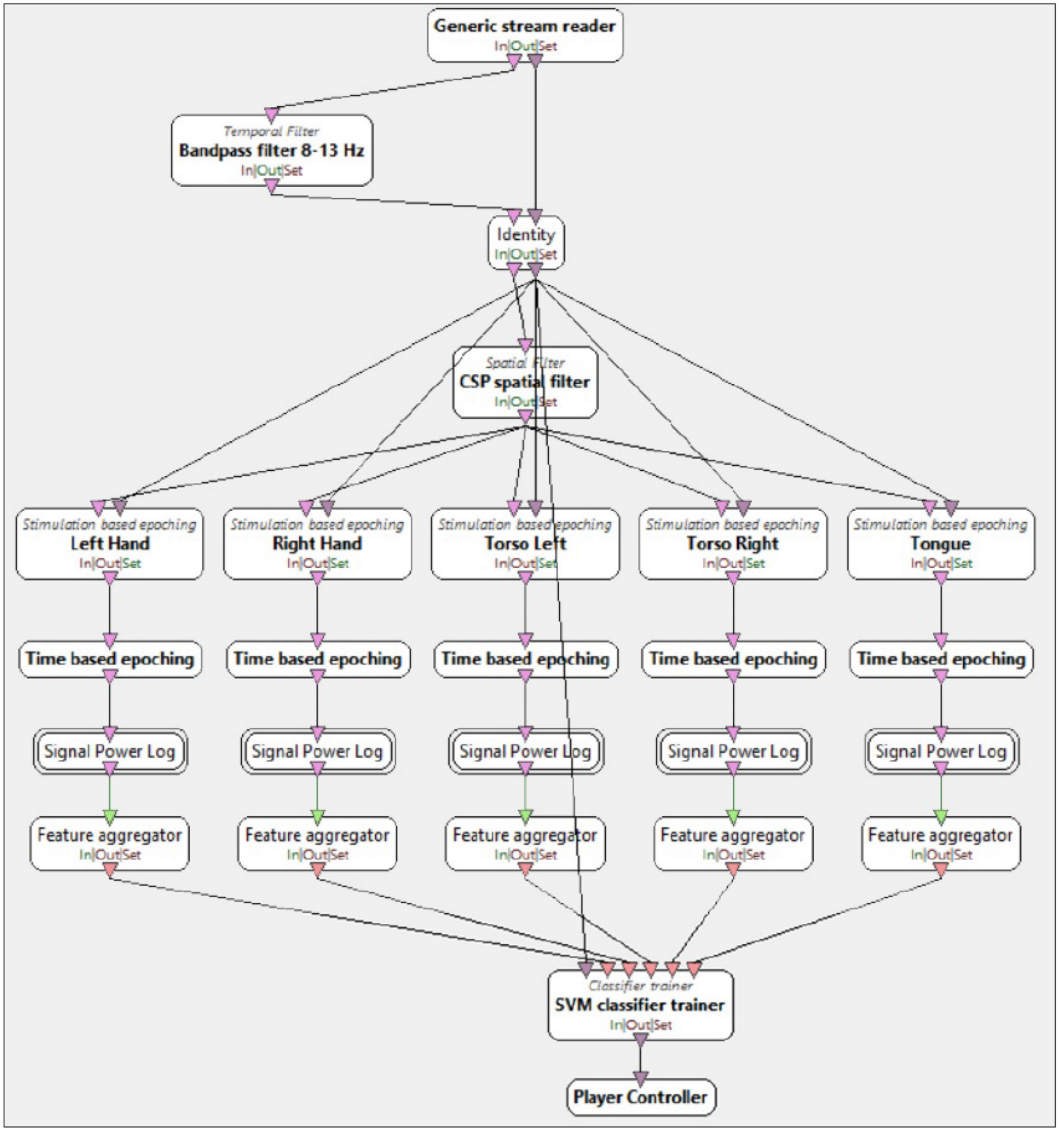
Screenshots of OpenViBE support vector classification (SVC) Trainer algorithm. Combination of pipeline steps for training the SVC algorithm consisting of following steps: *Bandpass filter 8–13 Hz* Box, *common spatial pattern (CSP) spatial filter* box, *Stimulation based epoching* boxes (*Left Hand, Right Hand, Torso Left, Torso Right, Tongue*), T*ime based epoching* box, *Signal Power Log* box, *Feature aggregator* box and *SVM classifier trainer* box.

After training the CSP, the SVC classifier was trained. As illustrated in Figure 6, the Pipeline OpenViBE SVC Trainer consisted of the following components:

- bandpass filter 8–13 Hz cf. *Temporal filter* box named *Bandpass filter 8–13 Hz*,
- pretrained CSP filter cf. *Temporal filter* box named *CSP spatial filter*, which used the trained data from the previous step, i.e., OpenViBE CSP Trainer in Figure 5,
- division of data into 5 classes corresponding to the imagery movements cf. *Stimulation based epoching* boxes named accordingly to the imagery movement task performed by participants of this research: *Left Hand, Right Hand, Torso Left, Torso Right, Tongue*,
- the creation of epochs cf. *Time based epoching* box,
- power extraction cf. *Signal Power Log* box,
- features extraction cf. *Feature aggregator* box, and
- training of the SVC algorithm cf. *Classifier trainer* box named *SVM classifier trainer*.

The parameters selected for the SVC were based on the optimal settings obtained through GridSearch for set no. 4, which comprised the following 10 electrodes: T7, P3, FC5, F3, AF3, AF4, F4, FC6, P4, and T8.

**Figure 7:**
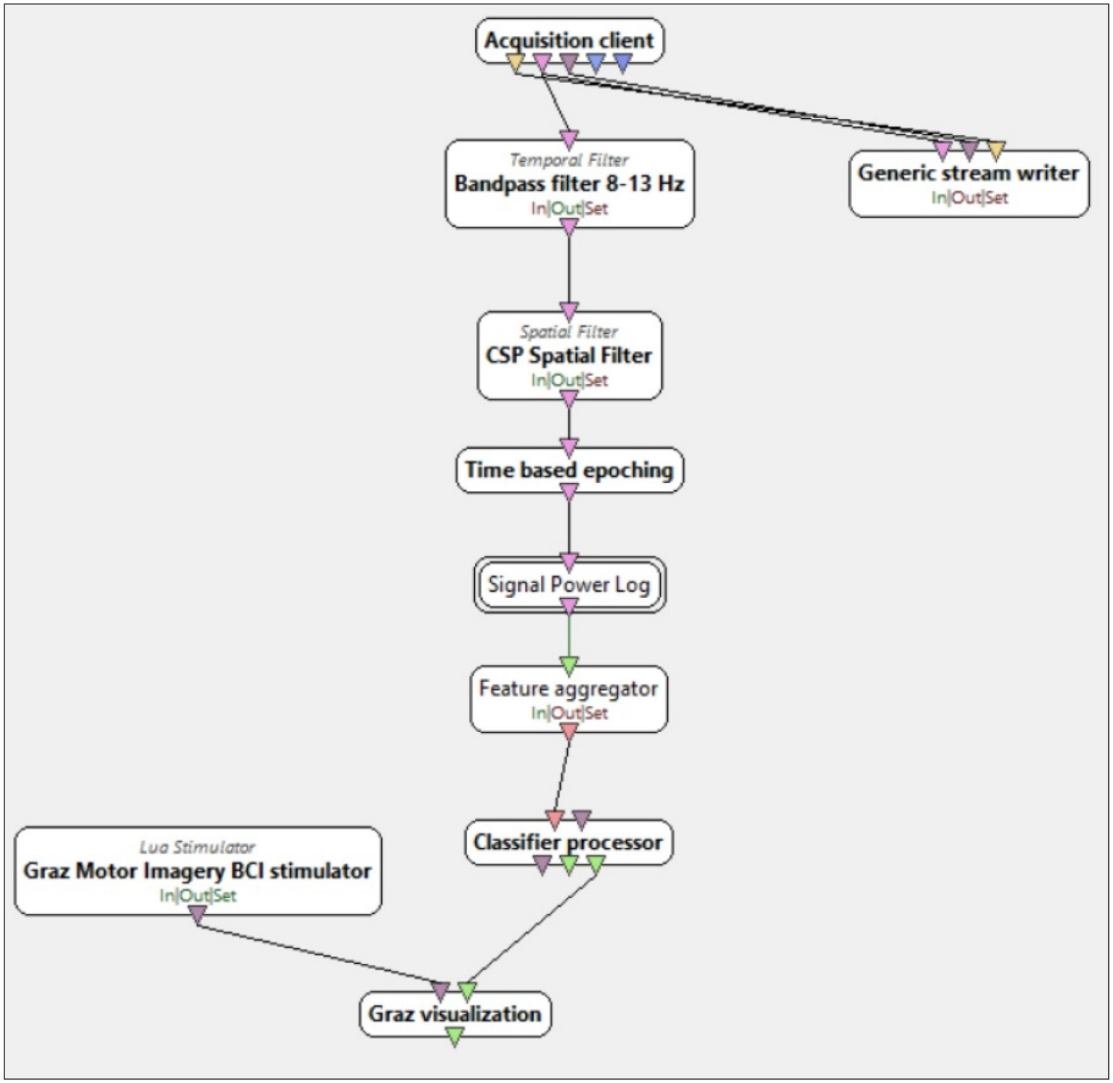
Screenshots of OpenViBE User Training/Testing algorithm. Combination of pipeline steps for user training and user testing consisting of the following steps: *Bandpass filter 8–13 Hz* Box, *CSP spatial filter* box, *Time based epoching* box, *Signal Power Log* box, *Feature aggregator* box, and textit Classifier processor box.

These steps were carried out offline. Finally, in the part of the study where the participant was asked to play “Soft Kitty” using only their thoughts, i.e., user training and user testing, the classification was performed online on previously trained CSP and SVC algorithms. The Pipeline OpenViBE User Training/Testing consisted of the following components, cf. Figure 7:

- bandpass filter 8–13 Hz cf. *Temporal filter* box named *Bandpass filter 8–13* Hz,
- pretrained CSP cf. *Temporal Filter* box named *CSP spatial filter* box), which used the trained data from the previous step, i.e., OpenViBE CSP Trainer,
- the creation of epochs cf. *Time based epoching* box,
- power extraction cf. *Signal Power Log* box,
- features extraction cf. *Feature aggregator* box, and
- pretrained SVC algorithm cf. *Classifier processor* box, that used the trained data from the previous step, i.e., OpenViBE CSP Trainer.

In all of the aforementioned pipelines, the signal was not filtered at the current frequency, as the *Emotiv EPOC X* is equipped with built-in digital notch filters operating at 50 Hz and 60 Hz applied to the raw data.

### 3.8 Semi-structured interview

To gain insight into the subjective experience of interacting with the developed BCI and to further investigate the impact of body awareness training on MI skills, a semi-structured interview was conducted with each participant after the study, detailed interview questions cf. Supplementary material. The interview questions addressed several topics, including the general comments of the participants, their approach to imagining movements, the ease and intuitiveness of MI, the perceived effectiveness of training, limitations related to memory and attention, feelings of mental and physical fatigue, the general participation in the study, motivation levels, clarity of instructions, understanding and usefulness of feedback, strategies for managing negative feedback and feelings of control and ownership over actions mediated by BCI.

### 3.9 Procedure

The procedure consisted of the following steps:

1. Signing the form of informed consent and consent to t.
2. Introducing the participant to the subject of the study, including the trial setup of the EEG set and showing the participant's EEG waves on the screen to make the participant aware that he has a volitional influence on changing his own EEG signal.
3. Pre-training conducting a questionnaire—Polish version of the MIQ-3—Movement Imagery Questionnaire (Budnik-Przybylska et al., 2016).
4. Body awareness training.
5. Post-training conducting a questionnaire—Polish version of the MIQ-3—Movement Imagery Questionnaire (Budnik-Przybylska et al., 2016),
6. data acquisition.
7. Classifier training.
8. User training—preparing the participant to play the melody.
9. User testing—playing the “Soft Kitty” melody.
10. Conducting a semi-structured interview.

The duration of the study for one participant is 2.5 h. Data from each stage where the participant performed MI tasks, i.e., data acquisition, user training, and user testing, were recorded.

## 4 Results

Results are organized into four main categories: motor imagery ability, classification performance of machine learning models, EEG data findings, and participants' subjective evaluations. Each category highlights the impact of body awareness training, the effectiveness of the classification approach, observed neural activity during motor imagery, and user feedback on the overall study experience.

### 4.1 Results of movement imagery questionnaire-3

Table 3 presents the mean, standard deviation, skewness, and kurtosis for both the pre-training and post-training conditions across the visual and kinesthetic subscales. In both conditions, participants rated their visual MI higher than their kinesthetic MI, demonstrating that visual MI was easier for participants to perform. A negative skewness score suggests a rightward shift in the distribution, indicating the above-average capability of the participants in imagining movements. In contrast, positive kurtosis values suggest that the results predominantly indicate high levels of motor image ability. An exception is noted in the kinesthetic subscale under the post-training condition, where skewness is close to zero, suggesting an average ability of participants to imagine movements in this context. Furthermore, visual inspection suggests a trend toward higher post-training scores, with an increase in average values observed for both subscales following body awareness training.

**Table 3:** Statistics and distribution for the subscales.

**Subscale**	***N***	**Mean**	**SD**	**Skewness**	**Kurtosis**
**Pre-training**					
Visual	7	5.64	0.95	-0.58	0.87
kinesthetic	7	5.00	1.19	-0.43	0.05
**Post-training**					
Visual	7	5.71	1.12	-1.10	0.89
kinesthetic	7	5.57	0.79	-0.01	-0.25

N, number of participants; SD, standard deviation.

The individual item analysis of the pre-training and post-training conditions is shown in Table 4. For pre-training conditions, visual MI was rated higher than kinesthetic MI, except for item 2, where kinesthetic MI for a specific movement was rated higher than item 1, where visual MI was used for the same movement. For the post-training condition, visual MI was rated higher than kinesthetic MI, except item 2, where kinesthetic MI for a specific movement was rated higher than item 1, where visual MI was used for the same movement. Comparing both conditions, it can be seen that most of the scores in both visual and kinesthetic subscales increased in the post-training condition.

**Table 4:** Individual item analysis for pre-training and post-training conditions.

**Subscale**	**Item**	**Pre-training**		**Post-training**	
		**Mean**	**SD**	**Mean**	**SD**
Visual	2	5.00	1.29	5.14	1.21
	4	5.86	0.69	6.00	1.00
	6	5.29	0.49	5.43	1.27
	8	6.43	0.53	6.29	0.76
kinesthetic	1	5.43	0.98	5.71	0.95
	3	4.71	1.70	5.43	0.98
	5	5.00	0.82	5.43	0.79
	7	4.86	1.21	5.71	0.49

The item column shows the number of individual mental tasks where the only difference between these tasks is that in the first mental task, the participant imagined the movement visually, while in the second, the exact same movement was imagined kinesthetically. SD, standard deviation.

### 4.2 Machine learning efficiency

The effectiveness of machine learning classification was evaluated using EEG data collected during the data acquisition, user training, and user testing phases. All calculations were performed offline. The evaluation metrics included training and test accuracy, Cohen's Kappa coefficient, confusion matrices, sensitivity, and specificity. These metrics were computed for two conditions: user training and user testing, and were calculated separately for each participant.

The classifier's accuracy for both user training and user testing conditions, calculated for each participant, is presented in Table 5. No signal was recorded during the first participant's user training condition due to a technical failure. The train accuracy in both conditions was similar for each participant, with a range of 0.48–0.60 for user training and 0.48–0.62 for user testing. A slight increase in test accuracy was observed in the user testing condition compared to the user training condition: test accuracy for user training ranged from 0.18 to 0.25, while for user testing it was between 0.17 and 0.36. The significant discrepancy between training and test accuracy in both conditions suggests overfitting, where the classifier performs well on known data, the training data, but poorly on unseen data (Ying, 2019).

**Table 5:** Classification results: Accuracy and Cohen's Kappa coefficient for each participant for user training and user testing conditions.

**ID**	**Train accuracy (user training)**	**Test accuracy (user training)**	**Cohen's Kappa (user training)**	**Train accuracy (user testing)**	**Test accuracy (user testing)**	**Cohen's Kappa (user testing)**
S01	—	—	—	0.53	0.25	0.02
S02	0.60	0.18	-0.02	0.62	0.31	0.12
S03	0.55	0.20	0.00	0.62	0.28	0.12
S04	0.57	0.22	0.02	0.57	0.36	0.19
S05	0.58	0.22	0.02	0.58	0.31	0.14
S06	0.48	0.25	0.06	0.48	0.17	-0.03
S07	0.50	0.22	0.02	0.50	0.19	-0.03

(—) not provided.

Cohen's Kappa coefficient was slightly higher than zero in most cases. This coefficient value indicates that most of the classifications performed by the classifier were close to or somewhat higher than the chance level. The exception here is the two results achieved for s06 and s07, where Cohen's Kappa is lower than zero for the user testing condition. The classification in these two cases was random.

### 4.3 EEG data analysis

EEG data analysis was performed to visualize event-related desynchronization (ERD) and event-related synchronization (ERS) of power for each imagined movement during the data acquisition stage. Since the signal processing pipeline used in classifier training relies on power computations from the OpenViBE Signal Power Log box, power change visualizations were created to complement classifier results. The analysis was carried out using MNE Python 1.4.2 (Gramfort et al., 2013), an open-source Python package, following the pipeline proposed by (Storti et al. 2016).

Due to the *Emotiv EPOC X* headset's incomplete coverage of the central scalp area, an extensive visual inspection of the data was first conducted to familiarize oneself with signals from unevenly distributed sources. Based on this inspection, previous experience, and EEG literature on *Emotiv EPOC X* data (Melnik et al., 2017; Mehmood and Lee, 2016), artifacts were identified and removed using independent component analysis (ICA). The signal was re-referenced to a standard average reference, and a 1–30 Hz bandpass filter was applied using the Parks-McClellan finite impulse response (FIR) filter.

From each EEG file, 60 time periods were selected, corresponding to 12 trials per imagined movement condition: left hand, right hand, left lateral bending, right lateral bending, and tongue movements. Each trial lasted 9 s. The baseline was set in the time window from -3.0 to -1.0 s before the cue, corresponding to the display of the fixation cross. For further analysis, a 3 s time window was selected from 2.5 to 5.5 s after cue presentation to avoid signal disruptions caused by saccades as participants shifted their gaze from the backlit key to the fixation cross; each selected time window was then segmented into consecutive 500 ms epochs.

A fast fourier transform (FFT) with a non-overlapping Hamming window was applied to each epoch for each electrode, followed by averaging across epochs for each condition and participant. Power spectral density (PSD) was computed for the alpha, i.e., 8–13 Hz, and lower beta, i.e., 14–20 Hz, frequency bands. The alpha band was selected to match the frequency range used in classifier training, while the beta band was included because of its potential to show complementary power changes relative to the alpha band (Pfurtscheller and Lopes da Silva, 1999). ERD and ERS, the related topographic maps, can be found in Supplementary material, where they were calculated as the percentage of decrease or increase in power relative to the baseline power, as shown in Equation 1. This equation is adapted from (Storti et al. 2016); (Pfurtscheller and Lopes da Silva 1999), where α is the alpha frequency band. The ERDs of the beta band and the ERSs of the alpha and beta bands were computed accordingly.


(1) $$\begin{array}{l} ERD^{\alpha }=\frac{PSD_{condition}^{\alpha }-PSD_{baseline}^{\alpha }}{PSD_{baseline}^{\alpha }}\cdot 100 \end{array}$$

### 4.4 Semi-structured interview analysis

Semi-structured interviews recorded and transcribed were analyzed using deductive thematic analysis (Braun and Clarke, 2006; Nowell et al., 2017), with the help of MaxQDA 2022 software (Kuckartz and Rädiker, 2019). Three main themes were identified and are described below: MI, study design, and subjective experience.

#### 4.4.1 Theme 1: motor imagery

This theme explores the MI abilities of the participants, aiming to understand how they imagined the movements, whether it was intuitive and easy, and the strategies they used to improve the performance of the BCI. It includes five codes: modality and perspective of MI, content of MI, strategies used to mitigate negative feedback, and ease and intuitiveness of MI.

##### 4.4.1.1 Code 1: modality and perspective of motor imagery

Most participants (4 out of 7 participants) imagined the movements kinesthetically and used first-person visual imagery when possible, e.g., hand movements and lateral bending. One participant noted difficulty in separating these two modalities, as they felt interconnected: “*it was hard for me to distinguish between feeling and seeing because in my mind it always overlaps and forms a coherent whole.”* Two participants reported using only kinesthetic MI. One described the experience as having two bodies: a real body and a “ghost body” that performed the imagined movements: “*[…] part of my body was invisible, and when I was supposed to imagine the movement of the hand, I imagined another ghost hand that was coming out of my arm and was performing the movement.”* One participant reported imagining movements in the kinesthetic and either first or third person visual way, depending on the movement being imagined: “*concerning the tongue movements, I imagined them from beside me, because I cannot see it myself [from the first person perspective]. Concerning the other movements, I imagined them from the first-person perspective, as if I were looking down at my body.”* Moreover, they mentioned sometimes confusing their body image with the body image of the person from the training movie: “*at some point I looked more like her [the person from the movie] than myself.”*

##### 4.4.1.2 Code 2: the content of motor imagery

During data acquisition, participants focused on key points of each movement (4 participants) or on the overall experience of performing the movement (3 participants). The key point was defined as the starting reference for MI. “*such a characteristic point that you could stick to and then go from it to the rest of this movement,”* that, while participants performed the real movements, was identified as the most felt and the best-remembered feeling, “*the points I felt the most or remembered the most.”* The characteristic points of movements mentioned by the participants were: muscle movement, muscle tension, muscle contraction, muscle fatigue, pain, numbness, tendon movements, touch of body to body, e.g., nails digging into hand, tongue touching teeth or lips, touch of clothes to body.

##### 4.4.1.3 Code 3: strategies used to mitigate negative feedback

During user training and testing, participants received feedback based on the accuracy of the classification. When negative feedback occurred, they were instructed to adjust their MI strategy. All participants modified their approach: four changed their focus to a different characteristic point, two increased movement pace, one switched from left to right body movement, one focused on memorizing details, one enhanced movement granularity, one concentrated more on the task, and one shifted focus to physical discomfort experienced during the movement.

##### 4.4.1.4 Code 4: easiness of motor imagery

Regarding the ease of MI, only one participant reported that all movements were equally easy to imagine. The most complicated movements to imagine were tongue movements (3 participants), lateral bending (2 participants), and hand movements (1 participant). The movement was deemed hard to imagine when there was no (1 participant) or only one characteristic point (1 participant), the movement was very complex (1 participant), or, in case of two participants that considered negative feedback as an indicator of subjective ease of imagery, there was too much negative feedback. The easiest movements to imagine were hand movements (3 participants), tongue movements (2 participants), and lateral bending (1 participant). As explained by two participants, a movement was easy to imagine when the participant had already encountered the movement in a non-experimental context, and if there was a well-discernible characteristic point that was felt while performing the movement.

##### 4.4.1.5 Code 5: intuitiveness of motor imagery

Regarding the intuitiveness of MI, three participants found all movements intuitive to imagine. Four participants indicated that hand movements were the most intuitive, while one participant found tongue movements and lateral bending to be the most intuitive. Conversely, lateral bending (3 participants) and tongue movements (2 participants) were reported as the least intuitive. The intuitiveness of a movement was influenced by its frequency in everyday life (4 participants) and its complexity (1 participant).

#### 4.4.2 Theme 2: study design

This theme addresses specific elements of the study, including instructions, fixation point, body awareness training, feedback, breaks, and technical comments. Each element is categorized as a separate code within the theme. This theme aims to evaluate the study design and the effectiveness of the body awareness training.

##### 4.4.2.1 Code 1: feedback

All seven participants confirmed their understanding of the feedback provided. Furthermore, three participants actively sought patterns within the feedback: “*for example when I tried to move my tongue, it [feedback] always showed my left hand and right hand at the same time, […], maybe I was thinking about direction or something,”* and three participants suspected that the feedback was random.

Three participants found the feedback helpful, while four perceived it as somewhat helpful. Two participants appreciated receiving both positive and negative feedback, as it indicated ongoing changes in their brain activity: “*it is better to have any feedback so that we know that our brain is working.”* However, three participants noted a lack of clarity in the negative feedback, which indicated that they needed to modify their strategy: “*it is helpful in that you have to try something different,”* but it did not inform participants whether the changes that they had applied in the used strategy of MI yielded any results, “*when I saw that the interface didn't really catch what I meant, I tried to go back to what was in those videos […] I don't really know with what effect,”* and it did not contain specific instructions on how to improve it, “*I couldn't draw from this information any hint on what I should have changed in order that the computer scores a hit.”*

Positive feedback motivated three participants, while one stated that negative feedback served as a motivator, highlighting the need for improvement to achieve better results. “*I felt pressure that I should try harder.”* Furthermore, the negative feedback resulted in a change of the used MI strategy (4 participants), distraction (2 participants), “*I started to think about the movement that was highlighted [as the negative feedback], and not the one that I should have initially thought about,”* frustration (1 participant), overcomplication of MI (1 participant), “*I was trying too hard at some point—trying to figure out how to make it work—and I may have overcomplicated it a bit,”* and a surprise that it is negative (2 participants), “*it surprised me sometimes that [the feedback] wasn't correct.”* Furthermore, two participants regarded negative feedback as a direct indicator of their subjective experience of the ease of performing MI for the respective movements.

##### 4.4.2.2 Code 2: instructions

All participants indicated that the instructions were clear. One participant specifically appreciated the understanding checks conducted by one of the researchers “*I liked that check-ups made by you, because I knew exactly that I understood everything.”*

##### 4.4.2.3 Code 3: fixation point

Participants were questioned about their eye movements during the interview without an additional eye-tracking method to analyze gaze patterns. During the data acquisition stage, all participants reported shifting their gaze to the cue, i.e., the backlit key, and then returning their gaze to the fixation point when it appeared on the screen. In the user training and testing stages, two participants kept their gaze on the key to be activated, while five participants directed their gaze toward the feedback and cue. Regardless of the approach, the participants confirmed that they did not consistently maintain their gaze on the fixation point.

##### 4.4.2.4 Code 4: body awareness training

The seven participants reported that body awareness training was beneficial. Training facilitated the development of MI for the specified movements and served as explicit instruction for the expected actions, as noted by two participants: “*the video showed me exactly what movements you are expecting from me. So that was cool because I knew exactly what was going to happen,”* that allowed the normalization of imagined movements, “*when it comes to tongue movements, I would not do such a thing myself. I would not think that it was possible to do such a thing. I certainly would not stand in front of a mirror and see what it looks like.”* Moreover, the training allowed participants to focus their attention on the movements while performing them (3 participants) “*it brought that awareness back to what was going on in the movement, because it [training] was not done automatically, but with an inquiry, what is going on?”*, identify characteristic points of movement for MI (5 participants) and, as a result, to better rehearse the given movement during MI (3 participants), “*it was only during this training that I realized that I could do such a movement, and the movement had already been repeated many times and MI was just a recapture from muscle memory,”* because of the newly created memory of the given movement, “*[training] helped with the freshness of recalling these movements later”*. However, as some participants previously mentioned, there was confusion between their own body image and the body image presented during the training.

##### 4.4.2.5 Code 5: technical aspects of the study

During the interview, participants provided feedback on the technical aspects of the study. Three participants felt that specific components, including instructions and training, were overly lengthy. Regarding the study environment, three participants reported feeling discomfort: one cited a lack of natural light in the cabin, while two others found the *Emotiv EPOC X* uncomfortable due to its tight fit to their heads.

Regarding the instructions, one participant noted that the phrasing of several statements felt awkward, stating, “*you do not say it that way.”* Additionally, one participant found the distinction between visual and kinesthetic MI inadequately explained, and another preferred to read the instructions independently rather than have them read aloud by the researcher.

All seven participants confirmed that the five body movements were easy to perform and that they did not have difficulty remembering how to imagine performing them. However, two participants mentioned that the fixation cross was not clearly visible, describing it as too thin and black against a dark gray background. Lastly, a participant indicated that they initially associated the red feedback color with an error.

#### 4.4.3 Theme 3: subjective experience

The theme reflects the subjective experience of the participants. This theme aimed to determine in what state the participants found themselves and how they felt about the BCI-mediated actions they performed. Therefore, the theme contains codes concerning the participant's state, i.e., attention, fatigue, motivation, and interest, as well as codes that regard the senses of agency and ownership over BCI-mediated actions.

##### 4.4.3.1 Code 1: attention

Four participants reported difficulties in maintaining focus during the study. The reasons for losing concentration included thinking about personal responsibilities, their relationship with one of the researchers, and the methodological aspects of the study. Furthermore, a participant noted that the study did not naturally engage their attention: “*I wouldn't call the study interesting as very auto-focusing because you had to maintain the focus yourself.”*

##### 4.4.3.2 Code 2: fatigue

All seven participants reported feeling fatigued during the study – either physically (3 participants), mentally (1 participant), or both (3 participants). Reported causes included the study's length (3 participants), lack of movement during BCI interaction (2 participants), boredom (1 participant), eye strain (1 participant), discomfort from wearing the *Emotiv EPOC X* (2 participants), and feeling time pressure (1 participant): “*I felt a bit of time pressure, such that I only have these* 4 *s for this one key to light up,”* feeling of frustration (1 participant), general tiredness as a result of poor night sleep (1 participant), and finally, lack of natural light in the study environment (1 participant). All participants reported that breaks helped them recover energy.

##### 4.4.3.3 Code 3: motivation and interest

All seven participants expressed interest in the study and motivation to perform well. Four participants reported general motivation, two were motivated by their friendship with one of the researchers, and one was driven by the potential to contribute to technology that could aid people with disabilities as an alternative to a mouse and keyboard. All participants were interested in BCI technology, particularly its potential to enable communication without physical movement. One participant also found the interactive nature of the study particularly engaging.

##### 4.4.3.4 Code 4: sense of agency and sense of ownership

The sense of agency was defined as the participant's perception of being the cause of an action, while the sense of ownership refers to the feeling of being the one experiencing the action (Gallagher, 2000, 2012). Based on these definitions, all seven participants reported experiencing only partial or low control over their actions: “*if you include me with some convulsions, then yes, [I controlled it],” and ownership of actions, “it is actually me, not a video, […] that 'me' component was there too.”* Participants ascribed to the computer some of the control: “*[the keys were controlled by] an algorithm or an artificial intelligence or whatever you call it,” and the ownership of actions, “I'm just not sure if sometimes the computer didn't do it.”* The feeling of control in three participants depended on feedback, i.e., when the feedback was positive, they felt control, and when it was negative, they did not. One participant reported that their feeling of control increased with the course of the study: “*and especially at the end [...] I really knew that when I wasn't thinking about any of these things [movements] at all, or even about my body, none of them [the keys] would turn on.”* Finally, three participants were acquainted with the principles of operation of a BCI: “*I was probably the trigger that started the process of starting the keys, but which key fired was no longer entirely dependent on what I was trying to think about,”* and the rules of operation of their brain activity: “*I do it but there's quite a lot of noise going on.”*

## 5 Discussion

Despite general brain activation patterns during MI, variations exist depending on factors such as the specific body part involved, type of movement, modality, perspective, and individual differences, both between participants and within the same participant over time. Therefore, individualized training for each participant is crucial to optimize speed and accuracy in real-time MI-based BCIs.

To our knowledge, no previous motor imagery study has combined lateral bending with tongue and bilateral hand movements, making the present protocol a novel contribution to the field. Critically, we introduced a dedicated body-awareness training session to enhance motor imagery precision, in which each target action–lateral bending, tongue, and left- and right-hand movements–was physically demonstrated and rehearsed prior to the imagery blocks. This structured somatic preparation stands in contrast to previous studies, where the content of imagined movements was often underspecified or left entirely to participant interpretation. By anchoring motor imagery in embodied experience, our approach aims to improve the specificity and consistency of imagery movements.

### 5.1 Machine learning

Overfitting is evident in user training and user testing conditions for each participant, as indicated by much higher train scores than test scores. This discrepancy may result from insufficient data to achieve proper classification performance. The dataset in this study includes only 7 participants. For each participant, 12 trials per class at 5 classes were recorded in the data acquisition stage, the training set. The first test set was recorded in the user training stage–the data acquisition results totaled 60 trials. For the user testing stage, the second test set, only 36 trials were collected across the classes. Furthermore, the training data were divided into five subsets (k-fold = 5), probably contributed to overfitting due to limited data. Increasing the dataset by recruiting more participants, conducting additional trials, adding random noise, or generating synthetic data based on the existing distribution could mitigate this issue (Ying, 2019). However, this was intentionally omitted to maintain a simplified pipeline suitable for future application in ecological research.

Furthermore, the EEG data suffered from a low signal-to-noise ratio due to the absence of artifact-removal techniques such as ICA. ICA is among the most effective techniques for removing ocular and other noise artifacts. However, to meet the study's primary goal of developing a low-cost, mobile, and plug-and-play BCI suitable for battery-powered systems and ecological applications, ICA was omitted from the online processing pipeline. Algorithm hyperparameters could be optimized through grid search to reduce overfitting and improve classifier performance. Another way to improve the classification accuracy is by selecting an appropriate classifier. Although the SVC was chosen based on previous analysis, algorithms such as LDA have also shown promising results for MI tasks.

Feature selection is another critical aspect to consider for future improvements. More features can increase model complexity without necessarily improving accuracy, as some may act as noise (Ying, 2019). Reducing the number of features can be achieved using filter methods such as K Best, which selects k features giving the best score, percentile, which selects the indicated percentage of features giving the best score, False Positive Rate test, which selects features with p-value below the alpha threshold in the FPR test, or wrapper methods such as forward selection, which adds features from the list, creating a list of features giving the best score, or backward elimination, which removes features from the complete list of features, stopping at the smallest subset giving the best score. The PCA-based feature extraction method can also help reduce the number of data dimensions. GridSearch selects the best classifier and the most optimal parameters, and the algorithm searching for the best set of features has not been used due to hardware limitations.

Moreover, the choice of an EEG headset limited the data quality, as it lacked electrodes in the somatosensory cortex, C3 and C4, which are essential for MI. Future studies could benefit from equipment such as the Emotiv EPOC Flex or OpenBCI, which includes these electrodes.

Finally, interindividual variability in the frequency of the alpha band should be taken into account. The default assumption of an alpha range between 8 and 13 Hz does not account for individual differences in alpha frequency, which can reduce the signal-to-noise ratio and introduce noise into the data. The interindividual alpha peak frequency (IAF) should be measured to address this. IAF represents the dominant rhythm within the 5–15 Hz range, typically determined during a resting state with eyes closed. The individual alpha band is then defined as 20% above and below the IAF, allowing for a more personalized and accurate interpretation of the signal (Doppelmayr et al., 1998).

### 5.2 Complementary EEG data analysis

An EEG data analysis of the percentage changes in the power in each condition compared to the power of ERD/ERS baselines was conducted to complement the classification results. The results of the analysis were visualized on topographic maps in the alpha and beta frequency bands. No visible patterns of change were detected. The primary explanation for this observation is that ERD/ERS is generally identified over the central electrodes, specifically C3 and C4, which are absent in the *Emotiv EPOC X* configuration. Other factors that may have disturbed the changes in the EEG power distribution are participants' eye movements, fatigue, and problems with focusing their attention during the study. Therefore, the topographic representations of the changes in the power distribution are not the same for the *Emotiv EPOC X* and the headsets that possess electrodes over the central electrodes. For comparison, it was referred to other studies that have presented topographic maps of the changes in power during MI measured using the *Emotiv EPOC X*, e.g., (Kline and Desai 2014); (Martín-Chinea et al. 2019); (Soman et al. 2012).

Since the signal processing used to classify the incoming EEG data was based on power computation, the variability in the results may have been due to insufficient differentiation in power distributions across the scalp between different conditions. Additionally, within-condition power distributions may not have been consistent enough for the classifier to distinguish between them correctly.

### 5.3 An examination of study design and motor imagery ability considering participants' subjective experiences

The semi-structured interview provided valuable information on study design, body awareness training, and participants' MI abilities and subjective experiences.

In general, the participants confirmed that they understood the instructions and feedback. The interview revealed individual preferences on how instructions were presented and expressed. Training and feedback were generally considered helpful, and participants found the MI tasks to control the BCI easy to perform and remember. However, some participants felt that the study was too long, the environment lacked natural light, and the *Emotiv EPOC X* headset caused discomfort.

In this thesis, five body movement cues were presented via a five-key piano interface, and gaze-shift patterns associated with the piano's operation were identified during participant interviews. These patterns suggest that eye movements were involved, which could affect classification results. To mitigate this, signal processing and classification algorithms need an ocular artifact rejection step to avoid the direction of gaze that influences predictions. This adjustment would ensure that predictions are based on motor cortex activity rather than eye movements. However, such eye movements are common in BCIs for able-bodied users, where multimodal distractions, such as carting games or drone control, may affect performance (Schwarz et al., 2016).

Regarding feedback, consistent with the existing literature, e.g., (Roc et al., 2021), half of the participants noted that feedback could have been more responsive to changes in MI and provided more guidance on improving performance. The participants found the feedback motivating; half were driven by positive feedback, and the other half were driven by negative feedback. Although most BCI studies focus on the impact of biased feedback on motivation, cf. (González-Franco et al. 2011); (Mladenović 2020), this study did not use biased feedback or conduct psychological evaluations, so its influence on motivation remains speculative.

Some participants also confused feedback with the subjective difficulty of performing MI, interpreting feedback rates as an indicator of their performance. Similar responses have been observed in joystick-based teleoperation of robots (Mavridis et al., 2015), but this effect has not yet been fully explored in BCI research. These responses are problematic, as feedback in BCI systems typically reflects classification accuracy, which depends on factors such as signal processing and the chosen classifier, and not directly on the participant's MI abilities (Lotte and Jeunet, 2018). Furthermore, in line with previous findings that continuous feedback in MI-based BCIs can cause distractions (Schreuder et al., 2012; Schwarz et al., 2016), some participants in this study reported being distracted by the feedback.

The lack of informative and sensitive feedback, combined with its impact on the motivation of the participants and their perceived difficulty, highlights the need for an intelligent tutoring system for BCIs (Jeunet et al., 2016) and adaptive training procedures (Roc et al., 2021). Such systems should account for individual differences in learning and offer more personalized and responsive feedback.

Regarding participants' subjective experiences, the body awareness training helped standardize the imagined movements, allowing for a clearer study of MI experiences. Participants reported using kinesthetic or visual and kinesthetic imagery, focusing on specific points or holistic movement. They also imagined not only moving body parts and associated sensations, but also elements such as clothing, discomfort, and other body parts involved in movement. Consistent with previous research cf. (Roc et al. 2021), familiar to participants, simple, and with identifiable characteristic points were considered easier and more intuitive.

All participants generally considered training helpful, and they reported that it helped them focus on body movements, provided instruction on how to perform the movements, and helped create MI. However, some participants mentioned distractions, such as focus on clothing movement, prolonged movement discomfort, or other body parts involved. One participant also reported confusing their own body with the trainer's, suggesting potential negative effects on MI. In addition, training was perceived as too long.

Training was partially validated using the MIQ-3 questionnaire, with improvements observed in both kinesthetic and visual MI scores, especially on the kinesthetic subscale. However, these results should be interpreted cautiously due to the small sample size (7 participants), which limited statistical testing. Furthermore, repeated use of MIQ-3 could have contributed to improvement, rather than the training itself.

More validation of the training is needed to assess its impact on user performance and classification accuracy. Before conducting this validation, training should be improved by clearly explaining kinesthetic MI, explicitly instructing participants to engage in it, specifying the body areas to focus on for each movement, and encouraging attention to bodily sensations and movements.

Participant feedback indicated mental and physical fatigue, with some reporting difficulty focusing during the study-factors likely affecting classification accuracy. Although scheduled breaks were initially excluded to limit the session to 2.5–3 h, future protocols should incorporate brief, regular breaks—i.e., 3–5 min every 30–40 min—to reduce fatigue, improve comfort, and enhance data quality, even at the cost of slightly longer sessions.

Regarding the sense of agency in BCI-mediated actions, participants reported feeling limited, partial control, and ownership over the actions, likely due to the variability in the classification results. However, the interview was not detailed enough to fully explore the participants' sense of agency, which warranted further research.

The semi-structured interview used in this study was based on questions drawn from the literature on BCI user training, subjective experience in BCI-mediated actions, and user experience (UX) studies. However, UX methods in BCI research have been advocated (van de Laar et al. 2011, 2012), but they remain underutilized. The interview developed here could serve as a step forward in incorporating UX into BCI studies and should be further refined. Future improvements could include iterative additions to cover relevant aspects of BCI use, integrating simpler questionnaire-based questions on motivation and interest to encourage honest responses, explicitly defining terms like difficulty, intuitiveness, and philosophical concepts related to BCI actions, and refining questions associated with the sense of agency and responsibility.

### 5.4 Limitations

The hardware employed in this study, specifically the *Emotiv EPOC X* headset, posed significant limitations. The headset is a commercial EEG device that lacks central electrodes typically used in MI research, is available only in one size, operates via Bluetooth, and utilizes saline electrodes, all of which contribute to suboptimal signal quality. Consequently, these factors hindered the study's overall data quality and analysis capabilities.

Regarding the study design, the sample size of seven participants was insufficient for conducting robust statistical analyses of the collected data. Furthermore, the five-key piano application introduced ocular artifacts that were not mitigated during the online BCI process, potentially impacting the accuracy of the results. Moreover, the body awareness training utilized in the study had not been previously validated, which was limited by laboratory time and human resources. Lastly, the measures used for validation during the study were subjective, and repeated use of the MIQ-3 questionnaire may have influenced its results.

## 6 Conclusion

The conducted study demonstrated that extending the number of classes in an MI-based BCI utilizing the *Emotiv EPOC X* by replacing foot movement imagery with lateral bending movements, both left and right, and combining these with resting state, as well as imagery of left and right hand movements and tongue movements, is not feasible. The power changes in the EEG signals recorded with the *Emotiv EPOC X* during these MI tasks did not show sufficient differentiation for the employed SVC, enhanced by CSP-based feature extraction, to accurately distinguish between these states of brain activity.

A primary limitation contributing to this lack of differentiation is the absence of central electrodes in the *Emotiv EPOC X*, which impedes the precise capture of signal changes associated with MI. This limitation highlights the importance of utilizing equipment that can provide more comprehensive coverage of the motor cortex.

Despite these findings, the approach of combining these specific movements has not been previously explored, suggesting a potential avenue for future research. It is recommended to investigate whether alternative EEG headsets equipped with central electrodes can provide better performance for this type of BCI application, thus facilitating a more nuanced analysis of MI and expanding the capabilities of BCIs in practical settings.

## Data Availability

The raw data supporting the conclusions of this article will be made available by the authors, without undue reservation.

## Footnotes

^1^English equivalent of a popular Polish children's song “Wlazł kotek na płotek” https://en.wikipedia.org/wiki/Soft_Kitty; https://pl.wikipedia.org/wiki/Wlazl_kotek_na_plotek.

^2^Currently available kits include Muse https://choosemuse.com, NeuroSky https://neurosky.com, OpenBCI https://openbci.com, and Emotiv https://www.emotiv.com.

^3^https://processing.org in version 4.2.

^4^http://openvibe.inria.fr in version 3.5.0.

^5^Available at: http://gigadb.org/dataset/view/id/100295.
