# Supplementary material for: Motor imagery-based brain-computer interfaces: an exploration of multiclass motor imagery-based control for Emotiv EPOC X
Source: Front Neuroinform. 2025 Aug 12;19:1625279. doi: 10.3389/fninf.2025.1625279 (PMC12378764; doi:10.3389/fninf.2025.1625279)
Supplement: Supplementary file 1 [file Data_Sheet_1.pdf]

Supplementary Material

1 BODY AWARENESS TRAINING SCRIPT

Transcription of Body awareness training, translated from Polish to English.

The meditation training is designed to help you focus on your body, its movements, and the sensations that accompany them. Make sure you are in a quiet and safe place. Relax. Loosen your clothing. Sit comfortably and close your eyes. For the next several minutes, follow my voice with your thoughts. When other thoughts arise in your mind, acknowledge their presence and allow them to pass. During the training, I will ask you to perform a few exercises. As you do these exercises, allow yourself to focus on your body.

Start the training by focusing on your breath. Take a deep breath in through your nose and slowly exhale through your mouth. Take a deep breath in through your nose and slowly exhale through your mouth. Feel the air flowing through your body. As it enters through your nose, flows through your throat, and fills your lungs. Feel it staying there for a moment and then slowly returning, flowing through your throat and out through your mouth. Focus on your breath. Take a deep breath in through your nose and slowly exhale through your mouth. Calm your mind by staying focused on your breath. If you notice that your mind has wandered, return your focus to your breath.

Next is an exercise where you will focus on specific parts of your body. Keep your eyes closed during the exercise. Follow my instructions. Try to bring your attention to your physical sensations. Focus on feeling your body, its movements, and the sensations that accompany them.

Focus your attention on your feet. Notice how they rest. Feel their position. You can gently move them to feel them better. Do you feel how your feet are positioned? How they touch the surface they rest on? What do you feel when you focus on them?

Shift your attention to your calves. Focus on feeling them. What sensations do you experience? To help you focus on your calves, you can gently tense them and then relax them after a moment. Do you feel their presence? What sensations do you experience?

Now, focus on your thighs. On how they are positioned. Gently tense them. Do you feel them tighten? Relax the tension. Stay focused on your thighs for a moment.

Shift your attention to your hips. To how they are positioned. To the sensations you have when you think about your hips. Do you feel your hips? What do you feel when you focus on them?

Shift your attention to your abdomen. You can try gently tensing it to feel it better. After a moment, release the tension. Do you feel your abdomen move? Or perhaps it remained still? What bodily sensations accompanied your focus on your abdomen?

Shift your attention to your back. Notice how it is positioned. How much space it occupies. To help focus on feeling your back, you can gently move it. Stop the movement. What do you feel when you focus on your back?

Now focus on your chest. Where is it located? How is it positioned? Does it move with each of your breaths? What movements does it make? Stay focused on your chest for a moment.

Shift your attention to your fingers. How are they positioned? Can you feel their placement? You can gently move them to feel them better. What do you physically feel when you focus on your fingers?

Now, focus on your hands. Can you feel their presence? Where are they located? In what position are they placed? Focus on the physical sensations of having your hands.

Shift your attention to your wrists. If needed, gently move them to feel them better. Stop the movement. Can you feel their position? Focus on feeling your wrists.

Shift your attention to your arms. Notice how they are positioned. Tense them for a moment and release the tension. Do you feel your arms? How are they positioned?

Now focus on your neck. Can you feel its presence? How is it positioned? Is it tense? Try to relax your neck. What sensations accompany your focus on your neck?

Shift your attention to your face. Focus on your lips. How do you feel them? You exhale air through them while breathing. Can you feel its flow?

Shift your attention to your tongue. Can you feel it in your mouth? How do you feel its position? Gently move it. Stop the movement. How do you feel your tongue?

Shift your attention to your nose. Air flows through it as you breathe in. What does it feel like? Focus on your nose for a moment.

Now, shift your attention to your eyes. They are closed. Do they sometimes move? Try not to move them. Can you feel their presence? What is that sensation like?

This part of the exercise is now complete. Do not open your eyes yet and remain focused on your breath. Take a deep breath in through your nose and exhale through your mouth. Try to do the exercise again, this time directing your attention on your own. Focus on the individual areas of your body. During the exercise, try not to make any additional movements. Do not tense or relax your body. The tensing of muscles and gentle movements were intended to help you focus on your body more easily. Now, I ask you to remain still and scan your body, pausing for a moment on each part. Remember that you are doing this exercise for yourself, to help you better focus on your body. Take a deep breath in through your nose and exhale through your mouth. Begin focusing on the individual parts of your body.

In a moment, we will move on to the next exercise. If you were unable to focus on each part of your body, do not try to rush to finish the exercise. Leave it at the point where it was interrupted and accept it as a closed stage of the training. Follow my voice. The exercise aimed to help you focus on your body and its sensations. Take a deep breath in through your nose and exhale through your mouth. We will now proceed to the next exercise.

In this part of the training, you will perform exercises that will help you better understand the movements of your body. In later stages of the study, researchers will ask you to imagine these movements. Try to focus on these movements and fully engage in performing them, so that imagining these movements later will be as accurate as possible.

Open your eyes. On the screen, you will see a researcher who will demonstrate the movements that I will ask you to perform. Try to perform these movements as shown on the screen. While performing the movements, focus on your body. Pay attention to how you perform the movements and what you feel as you do them.

To begin the exercise, stand up from your chair and stand with your feet slightly apart.

We will start the exercise with the movement of the fingers on your right hand. Raise your right hand so that you can easily clench and relax your fist. Relax your hand. Straighten and spread your fingers. Slowly

clench your fingers into a fist. Tuck your thumb under the other four fingers. Keep your hand clenched in a fist for a moment, then straighten and spread your fingers again.

Relax your hand. Continue performing the movement at a similar pace to the one shown on the screen.

Once you feel confident with the movement, focus on the action itself. How are your fingers positioned? Are only your fingers involved in the movement? Does the center of your hand remain still? Does your wrist move? While clenching and opening the fist, only your fingers should be moving. If you're unsure whether you're performing the movement correctly, watch the movement demonstrated on the screen and compare it with your own.

Once you're sure you're doing the movement correctly, focus closely on how your fist clenches and opens. Try to remember as many details of the movement as possible. Pay attention to how your fingers move. Notice how they bend and open.

Now close your eyes and focus on the sensations that accompany the opening and clenching of your fist. Feel how your fingers are positioned. Focus on the pressure they exert on your hand. Feel how they relax.

Open your eyes and stop clenching your fist. Let it rest motionless at your side. In your mind, imagine the motion of clenching and opening your right fist. Imagine the sensations that accompany this movement. How clearly can you imagine the movement? Do you feel the sensations that accompanied the actual motion? Is the pace of the movement maintained?

If you find it difficult to imagine the movement or the sensations that come with it, you can open your eyes again and clench and open your fist a few more times. While performing the movement, focus on your fingers' movement and the sensations that accompany it. After a period you feel is appropriate, return to imagining the movement.

When you feel that you can clearly imagine clenching and opening your fist, click the arrow pointing to the right side of the screen to proceed to the next exercise.

Now, we will move on to the finger movement exercise for your left hand. Raise your left hand so that you can freely clench and relax your fist. Relax your hand. Straighten and spread your fingers. Slowly clench your fingers into a fist. Tuck your thumb under the other four fingers. Keep your hand clenched for a moment, then straighten and spread your fingers again.

Relax your hand. Continue performing the movement at a similar pace to the one shown on the screen.

Once you feel confident with the movement, focus on the action itself. How are your fingers positioned? Are only your fingers involved in the movement? Does the center of your hand remain still? Does your wrist move? While clenching and opening your fist, only your fingers should be in motion. If you're unsure whether you're performing the movement correctly, watch the movement demonstrated on the screen and compare it to the one you are doing.

Once you're sure you're doing the movement correctly, focus closely on how your fist clenches and opens. Try to remember as many details of the movement as possible. Pay attention to how your fingers move. Notice how they bend and open.

Now close your eyes and focus on the sensations that accompany the opening and clenching of your fist. Feel how your fingers are positioned. Focus on the pressure they exert on your hand. Feel how they relax. Can you imagine it?

Open your eyes. Stop clenching your fist and let it rest motionless at your side. In your mind, imagine the motion of clenching and opening your right fist. Imagine the sensations that accompany this movement. How clearly can you imagine the movement? Do you feel the sensations that accompanied the actual motion? Is the pace of the movement maintained?

If you find it difficult to imagine the movement or the sensations that come with it, you can close your eyes again and clench and open your fist a few more times. While performing the movement, focus on your fingers' motion and the sensations that accompany it. After a time you feel is appropriate, open your eyes again and return to imagining the movement.

When you feel that you can clearly imagine clenching and opening your left fist, click the arrow pointing to the right side of the screen to move on to the next movement.

Now, we will practice side bends. We will start with a bend to the right. Raise your left arm up, as shown on the screen. Bend your right arm and place it on your hip.

Slowly perform a side bend and return to the starting position. Continue the movement at a pace similar to the one shown on the screen.

Once you feel confident in performing the movement, focus on your torso's movement. How is it positioned? Is only your torso involved in the movement? Does your left arm make any additional movements? Does your right arm move? Is your head moving? While performing the side bend, only your torso should be in motion. Your arms and head will shift, but the movement originates from your torso, so focus your attention on it.

If you're unsure whether you're performing the movement correctly, watch the movement shown on the screen and compare it to the one you're doing. Once you're sure you're performing it correctly, focus on your body's movement. Try to remember as many details of the movement as possible. Pay attention to how your torso moves, how it bends, and how it straightens.

Now close your eyes and focus on the sensations you experience as you bend your torso to the right. Do you feel your torso bending? Do you feel it straightening? What does it feel like? Can you imagine it?

Open your eyes, stop bending, lower your arms, and stand still. In your mind, visualize the movement of bending your torso to the right. Imagine the sensations that accompany this movement. How clearly can you imagine the movement? Do you feel the sensations that accompanied the actual movement? Is the pace of the movement maintained?

If you're struggling to vividly imagine the movement or the accompanying sensations, you can close your eyes again and perform a few more side bends to the right. While performing the movement, focus on your torso's motion and the sensations that accompany it. When you feel ready, open your eyes again and return to imagining the movement.

Once you feel you can clearly imagine the side bend to the right, click the arrow pointing to the right on the screen to proceed to the next movement.

Now, we will practice side bends to the left. Raise your right arm up as shown on the screen. Bend your left arm and place it on your hip. Slowly bend to the left and return to the starting position. Continue the movement at a pace similar to the one shown on the screen.

When you feel confident enough with the movement, focus on your body's motion. How is it positioned? Is only your torso involved in the movement? Is your right arm making any additional movements? Is your left arm moving? Is your head moving? While performing the side bend, only your torso should be

in motion. Your arms and head will move, but the movement itself is driven by your torso, so focus your attention on it.

If you're unsure whether you're performing the movement correctly, watch the movement on the screen and compare it to your own. Once you're sure you're doing it right, focus on your body's movement. Try to remember as many details as possible. Pay attention to how your torso moves. Notice how it bends and straightens.

Now close your eyes and focus on the sensations you feel when bending your torso to the left. Do you feel your torso bending? Do you feel it straightening? What does it feel like? Can you imagine it?

Open your eyes, stop bending, lower your arms, and stand still. In your mind, imagine the movement of bending your torso to the left. Imagine the sensations that accompany the movement. How clearly can you picture the movement? Can you feel the sensations that accompanied the actual movement? Is the pace of the movement maintained?

If you're having trouble vividly imagining the movement or the accompanying sensations, you can close your eyes again and perform a few more side bends to the left. While doing this, focus on your torso's movement and the sensations it creates. After a suitable amount of time, open your eyes again and return to imagining the movement.

Once you feel you can clearly imagine the side bend to the left, click the arrow pointing to the right on the screen to proceed to the next movement.

Next, we'll practice a tongue movement. Relax your jaw. Open your mouth and extend your tongue as shown on the screen. Slowly move your tongue to the right, touching the right corner of your lips, then move it to the left, touching the left corner of your lips, and finally return it to the center of your mouth. Continue the movement at a pace similar to the one shown on the screen.

When you feel confident enough with the movement, focus on how your tongue moves. How is it moving? Is only your tongue involved? Are your lips making any additional movements? Is your jaw moving? Are your teeth shifting? While performing tongue movements, only your tongue should be in motion. Your lips, jaw, and teeth may move slightly, but the tongue is responsible for the movement, so focus your attention on it.

If you're unsure whether you're performing the movement correctly, watch the movement on the screen and compare it to your own. Once you're sure you're doing it correctly, focus on your tongue's movement. Try to remember as many details of the movement as possible. Pay attention to how your tongue moves. Notice how it shifts from side to side, touching the corners of your mouth.

Now close your eyes and focus on the sensations you feel when moving your tongue. Can you feel your tongue moving? How it touches the corners of your mouth? What does it feel like? Can you imagine it?

Open your eyes, stop moving your tongue, retract it, and close your mouth. In your mind, imagine the tongue movement you just practiced. Picture the sensations that accompany the movement. How clearly can you imagine the movement? Can you feel the sensations that accompanied the actual movement? Is the pace of the movement maintained?

If you're having trouble vividly imagining the movement or the accompanying sensations, you can close your eyes again and perform a few more tongue movements. As you do, focus on your tongue's movement and the sensations it creates. After a suitable amount of time, open your eyes again and return to imagining the movement.

Once you feel you can clearly imagine the tongue moving to the right and left, click the arrow pointing to the right on the screen to proceed to the end of the training.

This concludes the meditation training. Inform the researcher that the training is complete.

2 USED SUBSCALES OF THE POLISH VERSION OF THE MIQ-3

Transcription subscales of the Polish version of the MIQ-3, translated from Polish to English.

The questionnaire concerns two methods that some people use to internally (”mentally”) perform body movements. These methods are particularly relevant for certain types of movement. The first method involves trying to create a visual image of the movement in the mind. The second involves internally feeling the movement without actually performing it. You will be asked to try both ways of mentally representing various movements and then assess how easy or difficult each method was for you. The focus is not on whether one method was better or worse but on your ability to perform this type of mental task for each movement. There are no right or wrong answers, nor are any responses considered better or worse than others.

Each of the statements below describes a specific movement or action. I will read each description aloud, and I will ask you to perform the described movement physically, as outlined. The movement should be done once. Then, return to the starting position as if preparing to repeat the movement. Depending on the requested type of imagery, you will either (1) create as clear and vivid a visual image as possible of the movement you just performed—from an internal, first-person perspective, as if you were performing and observing the movement through your own eyes—or (2) internally feel the movement you just performed, without actually moving. After mentally performing the task, you will rate how easy or difficult it was to do so. You will use a seven-point rating scale, where 1 means very difficult and 7 means very easy. After each movement, I will ask you to rate it as accurately as possible, taking as much time as you need to carefully evaluate each task. The same ratings may apply to different movements, and there is no need to use the entire scale.

1. Stand with your feet and legs together, arms by your sides. Lift your right knee as high as possible, while your left foot remains on the ground and your right leg bends at the knee. Then lower your right leg and return to standing on both feet. Perform this action slowly. Return to the starting position. Now, try to feel yourself performing the movement you just observed, but without actually doing it. Then, rate how easy or difficult it was to mentally perform this task. Use a scale from 1 to 7, where 1 means very difficult to feel, 2 means difficult to feel, 3 means somewhat difficult to feel, 4 means neither easy nor difficult to feel, 5 means somewhat easy to feel, 6 means easy to feel, and 7 means very easy to feel.
2. Stand with your feet and legs together, arms by your sides. Make a low bend at the waist, then jump as high as you can, extending both arms above your head. Land on the ground with your feet apart and your arms down at your sides. Return to the starting position. Try to visualize how you performed the movement you just observed—from an internal perspective. Then, rate how easy or difficult it was to mentally perform this task. Use a scale from 1 to 7, where 1 means very difficult to visualize, 2 means difficult to visualize, 3 means somewhat difficult to visualize, 4 means neither easy nor difficult to visualize, 5 means somewhat easy to visualize, 6 means easy to visualize, and 7 means very easy to visualize.

3. Stand with your feet slightly apart and your arms extended above your head. Slowly bend your torso forward at the waist until you can touch your toes (or, if possible, the floor with your fingers or palms). Return to the starting position and stand straight with your arms extended above your head. Assume the starting position. Try to feel yourself performing the movement you just observed, but without actually doing it. Then, rate how easy or difficult it was to mentally perform this task. Use a scale from 1 to 7, where 1 means very difficult to feel, 2 means difficult to feel, 3 means somewhat difficult to feel, 4 means neither easy nor difficult to feel, 5 means somewhat easy to feel, 6 means easy to feel, and 7 means very easy to feel.
4. Stand with your feet and legs together, arms by your sides. Lift your right knee as high as possible, keeping your left foot on the ground while your right leg bends at the knee. Then lower your right leg and return to standing on both feet. Perform this action slowly. Return to the starting position. Try to visualize how you performed the movement you just observed—from an internal perspective. Then, rate how easy or difficult it was to mentally perform this task. Use a scale from 1 to 7, where 1 means very difficult to visualize, 2 means difficult to visualize, 3 means somewhat difficult to visualize, 4 means neither easy nor difficult to visualize, 5 means somewhat easy to visualize, 6 means easy to visualize, and 7 means very easy to visualize.
5. Use your non-dominant hand and extend your entire arm out to the side so that it is parallel to the ground, with your palm facing downward. Move your arm forward until it is directly in front of your body (still parallel to the ground). The arm should remain straight throughout the movement. Perform the movement slowly. Return to the starting position. Try to feel how you performed the movement you just did, but without actually executing it. Then, rate how easy or difficult it was to mentally perform this task. Use a scale from 1 to 7, where 1 means very difficult to feel, 2 means difficult to feel, 3 means somewhat difficult to feel, 4 means neither easy nor difficult to feel, 5 means somewhat easy to feel, 6 means easy to feel, and 7 means very easy to feel.
6. Stand with your feet slightly apart and your arms extended above your head. Slowly bend your torso forward at the waist until you can touch your toes (or, if possible, touch the floor with your fingers or palms). Return to the starting position and stand straight with your arms extended above your head. Assume the starting position. Try to visualize how you performed the movement you just observed—from an internal perspective. Then, rate how easy or difficult it was to mentally perform this task. Use a scale from 1 to 7, where 1 means very difficult to visualize, 2 means difficult to visualize, 3 means somewhat difficult to visualize, 4 means neither easy nor difficult to visualize, 5 means somewhat easy to visualize, 6 means easy to visualize, and 7 means very easy to visualize.
7. Stand with your feet and legs together, arms by your sides. Make a low bend at the waist, then jump as high as you can, extending both arms above your head. Land on the ground with your feet apart and your arms down at your sides. Return to the starting position. Try to feel how you performed the movement you just did, but without actually executing it. Then, rate how easy or difficult it was to mentally perform this task. Use a scale from 1 to 7, where 1 means very difficult to feel, 2 means difficult to feel, 3 means somewhat difficult to feel, 4 means neither easy nor difficult to feel, 5 means somewhat easy to feel, 6 means easy to feel, and 7 means very easy to feel.
8. Use your non-dominant hand and extend your entire arm out to the side so that it is parallel to the ground, with your palm facing downward. Move your arm forward until it is directly in front of your body (still parallel to the ground). The arm should remain straight throughout the movement. Perform the movement slowly. Return to the starting position. Try to visualize how you performed the movement you just observed—from an internal perspective. Then, rate how easy or difficult it was to

mentally perform this task. Use a scale from 1 to 7, where 1 means very difficult to visualize, 2 means difficult to visualize, 3 means somewhat difficult to visualize, 4 means neither easy nor difficult to visualize, 5 means somewhat easy to visualize, 6 means easy to visualize, and 7 means very easy to visualize.

3 SEMI-STRUCTURED INTERVIEW

Semi-structured interview questions translated from Polish to English.

The interview will cover the entire study and will focus on your subjective experience, so there are no right or wrong answers. We conduct it to improve our interface. Please try to be as honest with us as possible. Before we begin, I would like to ask you a general question. Did any comments about the study come to your mind while you were performing it or after it was completed? Is there anything you noticed that you would like to share? If any comments or suggestions arise during the interview, feel free to share them.

- During the study, you were asked to imagine five movements. Could you please describe how you visualized them?
- Was any of these movements easier or harder for you to imagine?
- Was any of the movements less or more intuitive?
- Was it easy or difficult for you to remember how to perform the individual movements?
- The training (recording) aimed to help you visualize the specific movements. Do you think it fulfilled its purpose or not?
- Was it easy or difficult for you to maintain your gaze on the fixation point?
- Did you find the study interesting or not?
- Did you feel motivated to perform well during the study, or not?
- Did you experience fatigue during the study? What kind of fatigue was it? Did breaks help you regain energy?
- Were the instructions (general instructions at the beginning of the study, instructions regarding the questionnaire, instructions regarding the training, instructions regarding the interface) clear or not?
- Was the feedback provided by the computer understandable to you or not? Was it helpful?
- When you received negative feedback, what did you do to improve?
- Did you feel in control of the keys while performing the tasks?
- Did you feel that you were the one activating the keys while performing the tasks?

4 TOPOGRAPHIC MAPS

Topographic maps showing the changes in ERD/ERS for each subject in alpha and beta bands were computed for each participant in each condition and are presented in Table S1. There exist no visible patterns in the topographic representations across participants within the same frequency band and condition.

Table S1. Topographic maps representing ERD/ERS. Each topographic map represents a percentage change in the power spectrum in the 3-seconds time window from 2.5 to 5.5 seconds after the end of cue presentation, compared to baseline taken from -3 to -1 seconds berore cue presentation. The changes in power are presented in alpha (8-13 Hz) and lower Beta (14 – 20 Hz) frequency bands for each of the seven participants in each of the five conditions of motor imagery: left hand, right hand, left lateral bending, right lateral bending, tongue movements.

		Left hand	Right hand	Left bend	Right bend	Tongue
S1	α	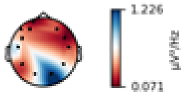	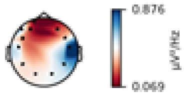	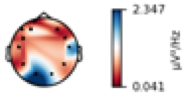	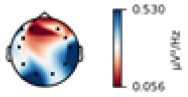	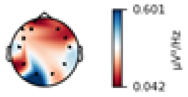
	β	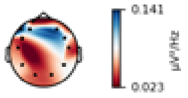	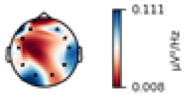	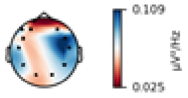	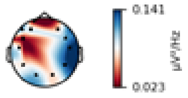	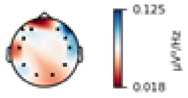
S2	α	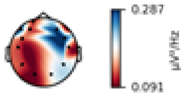	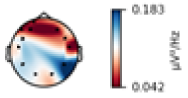	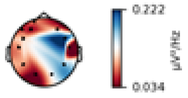	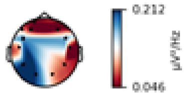	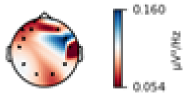
	β	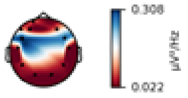	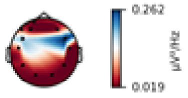	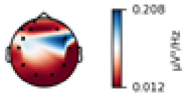	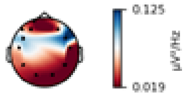	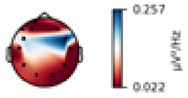
S3	α	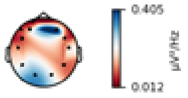	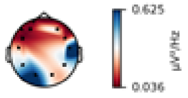	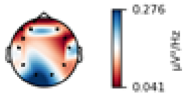	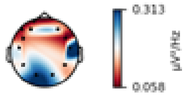	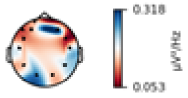
	β	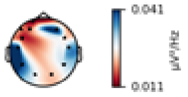	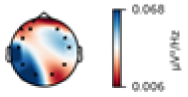	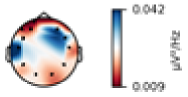	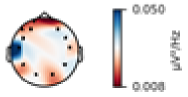	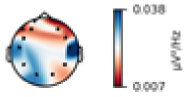
S4	α	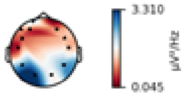	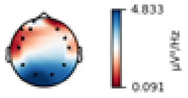	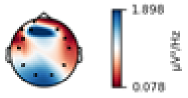	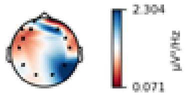	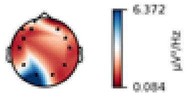
	β	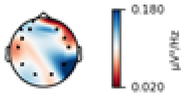	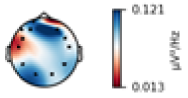	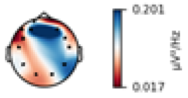	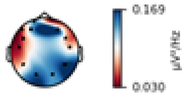	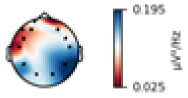

Table S1. Continuation of the table: Topographic maps representing ERD/ERS.

		Left hand	Right hand	Left bend	Right bend	Tongue
S5	α	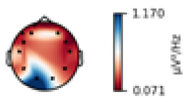	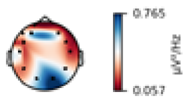	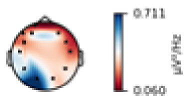	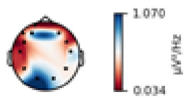	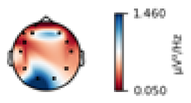
	β	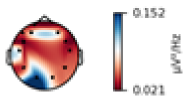	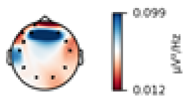	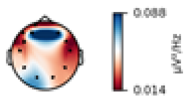	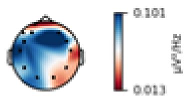	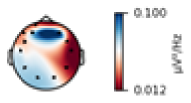
S6	α	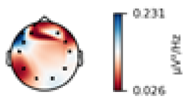	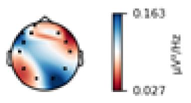	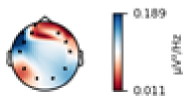	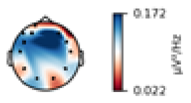	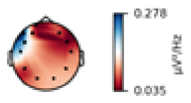
	β	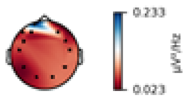	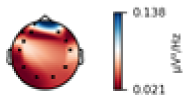	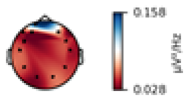	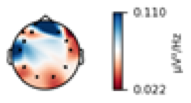	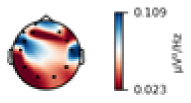
S7	α	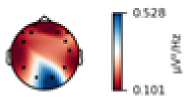	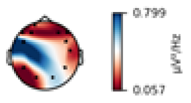	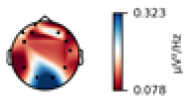	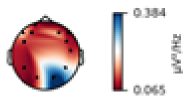	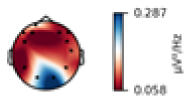
	β	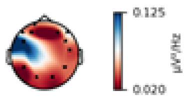	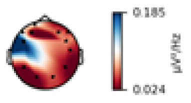	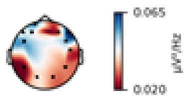	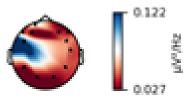	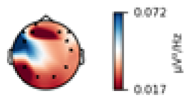
